# Strategies for communicating scientific evidence on healthcare to managers and the population: a scoping review

**DOI:** 10.1186/s12961-023-01017-2

**Published:** 2023-07-10

**Authors:** Rachel Riera, Carolina de Oliveira Cruz Latorraca, Roberta Carreira Moreira Padovez, Rafael Leite Pacheco, Davi Mamblona Marques Romão, Jorge Otávio Maia Barreto, Maria Lúcia Teixeira Machado, Romeu Gomes, Silvio Fernandes da Silva, Ana Luiza Cabrera Martimbianco

**Affiliations:** 1 Hospital Sírio-Libanês, Rua Barata Ribeiro, 142, 2O andar, São Paulo, SP 01308-000 Brazil; 2 Universidade Federal de São Paulo (Unifesp), São Paulo, Brazil; 3 Instituto Veredas, São Paulo, Brazil; 4 Fundação Oswaldo Cruz, Brasília, Brazil; 5 Universidade Metropolitna de Santo (Unimes), Santos, Brazil; 6 Universidade Federal de São Carlos, São Carlos, Brazil

**Keywords:** Knowledge translation, Scientific communication, Evidence-based informed policy

## Abstract

**Background:**

Health evidence needs to be communicated and disseminated in a manner that is clearly understood by decision-makers. As an inherent component of health knowledge translation, communicating results of scientific studies, effects of interventions and health risk estimates, in addition to understanding key concepts of clinical epidemiology and interpreting evidence, represent a set of essential instruments to reduce the gap between science and practice. The advancement of digital and social media has reshaped the concept of health communication, introducing new, direct and powerful communication platforms and gateways between researchers and the public. The objective of this scoping review was to identify strategies for communicating scientific evidence in healthcare to managers and/or population.

**Methods:**

We searched Cochrane Library, Embase®, MEDLINE® and other six electronic databases, in addition to grey literature, relevant websites from related organizations for studies, documents or reports published from 2000, addressing any strategy for communicating scientific evidence on healthcare to managers and/or population.

**Results:**

Our search identified 24 598 unique records, of which 80 met the inclusion criteria and addressed 78 strategies. Most strategies focused on risk and benefit communication in health, were presented by textual format and had been implemented and somehow evaluated. Among the strategies evaluated and appearing to yield some benefit are (i) risk/benefit communication: natural frequencies instead of percentages, absolute risk instead relative risk and number needed to treat, numerical instead nominal communication, mortality instead survival; negative or loss content appear to be more effective than positive or gain content; (ii) evidence synthesis: plain languages summaries to communicate the results of Cochrane reviews to the community were perceived as more reliable, easier to find and understand, and better to support decisions than the original summaries; (iii) teaching/learning: the Informed Health Choices resources seem to be effective for improving critical thinking skills.

**Conclusion:**

Our findings contribute to both the knowledge translation process by identifying communication strategies with potential for immediate implementation and to future research by recognizing the need to evaluate the clinical and social impact of other strategies to support evidence-informed policies.

*Trial registration* protocol is prospectively available in MedArxiv (doi.org/10.1101/2021.11.04.21265922).

**Supplementary Information:**

The online version contains supplementary material available at 10.1186/s12961-023-01017-2.

## Contributions to the literature

- Communicating results of scientific studies, effects of interventions and health risk estimates, in addition to understanding key concepts of clinical epidemiology and interpreting evidence, account for a set of essential instruments to bridging the gap between science and practice.
- There is still a lack of understanding about the exact types of strategies for communicating scientific evidence.
- The findings of this manuscript, specifically the extensive and detailed list of strategies mapped out to communicate scientific evidence in health to managers and the population, may support the decision-making of stakeholders involved in the process of knowledge translation within the realms of evidence-informed policies.

## Introduction

Within the context of evidence-informed policy (EIP), evidence syntheses should provide scientifically based information on health conditions, interventions, procedures, policies and programmes to meet the needs of health professionals, patients and public or private health managers. However, evidence obtained from scientific studies, especially from systematic reviews, other syntheses of multiple studies and clinical trials, is complex and often difficult for the general public to comprehend [1]. Health evidence needs to be communicated and disseminated in a manner that is clearly understood by decision-makers, especially in settings that demand rapid responses.

As an inherent component of health knowledge translation, communicating results of scientific studies, effects of interventions and health risk estimates, in addition to understanding key concepts of clinical epidemiology and interpreting evidence, represent a set of essential instruments to reduce the gap between science and practice. These needs have represented a challenge within the EIP worldwide.

Strategies for communication of evidence in health have the initial goal of increasing the understanding of the results of scientific research and should cover products, actions and approaches aligned to the needs of the manager (facing the demands for healthcare services) and the population (reliable information based on the best scientific evidence available) [2]. Nevertheless, the ultimate expected outcome of any effective communication, addressed to specific audiences, would be its clinical benefit (when considering individual health) and/or positive impact on health systems and organizations (when considering public health).

In this sense, expanding investment and improving skills in communication enables the identification of the best strategies to be used to overcome the barrier between evidence in health and managers and the population. Clear communication and active dissemination of health evidence to all relevant audiences in an understandable and accessible manner are essential to raise awareness of the importance of using scientific evidence, to support individual and population health-related decisions [1], and contribute to adherence to behaviours associated with positive health outcomes.

In the last decade, the advancement of digital and social media has reshaped the concept of health communication, introducing new, direct and powerful communication platforms and gateways between researchers and the public [2–4]. Various strategies such as plain language summaries and infographics have been devised and experimented for this purpose.

Some synthesis of strategies for communication of scientific evidence are available in the literature, including overviews of systematic reviews that address knowledge translation and general health communication strategies (for the population, health professionals and managers) [2, 5], systematic reviews restricted to communicating health benefits/risks [6–8] or teaching/learning strategies [9], narrative reviews [10, 11] and scoping reviews focused on communicating uncertainties [12]. No in-depth scoping review was identified with the objective of identify strategies for communicating scientific evidence on healthcare to managers and the population.

Thus, a mapping is necessary, through a scoping review, to identify the available strategies for communication of scientific evidence; the characteristics, barriers and facilitators for its implementation; the target audience and the context, as well as the gaps in the literature about its impact on healthcare. The results identified may constitute a valuable instrument for decision-making for sectors involved in promoting the use of scientific knowledge in decision-making processes related to the communication of evidence in the context of EIP.

## Methods

### Design and setting

This scoping review is a component of the project Apoio à Formulação e Implementação de Políticas Públicas de Saúde Informadas por Evidências (ESPIE), triennium 2021/2023, conducted at the Hospital Sírio-Libanês (São Paulo, Brazil), within the scope of the Programa de Apoio ao Desenvolvimento Institucional do Sistema Único de Saúde (PROADI-SUS), in partnership with the Department of Science and Technology of the Secretariat of Science, Technology, Innovation and Strategic Inputs of the Ministry of Health. This review was planned and conducted according to the recommendations of the Joanna Briggs Institute Manual for scoping reviews [13].

The report of the review followed the recommendations of the Preferred Reporting Items for Systematic Reviews and Meta-Analyses – extension for scoping reviews (PRISMA-ScR) [14]. The review protocol was planned and prospectively made available in the MedRxiv pre-prints database. [15]

### Methods for engaging the community and other stakeholders in the review

Stakeholder consultation was carried out throughout the development of the protocol, with the aim of increasing the applicability of its results and supporting the communication and translation of its results to the community. To this end, the following stakeholders were informally consulted: consumers (managers, health professionals and patients), experts in ‘knowledge translation’ and ‘health communication’ and information specialists.

### Criteria for inclusion of studies

The question of interest for this review was structured using the acronym PCC, which then guided the eligibility criteria as follows:

- P (population, condition): health managers and the general population.
- C (concept): strategies for communicating scientific evidence to health managers and/or the community. In this review, scientific evidence was considered as information obtained from the results of scientific studies and used to support or refute a health recommendation or the planning of health systems and policies. Thus, strategies were considered as those aiming to translate scientific and/or methodological information in a format/content geared to ensure the understanding of health managers and society of terms, criteria, tools and approaches related to scientific evidence in health. Any strategy focused on the communication of scientific evidence for this target audience was considered, including, for example, communication strategies to support health managers in decision-making, communications used during the organization of services and/or health systems, communication strategies to encourage the use of scientific evidence in the decision-making process, to increase access to health information from the perspective of the population, strategies for adapting the knowledge obtained by evidence to the local context, and so on. Studies on individual professional–patient communication (including diagnosis, communication of bad news and specific recommendations on individual therapy or prevention, among others) or specific to a particular health condition were not considered. Studies addressing the process of knowledge translation were included only when they reported, implemented and/or evaluated strategies for communication of scientific evidence as part of this process. Studies specifically addressing evidence dissemination and implementation strategies were not included.
- C (context): individual or public health; within public, private or supplementary health systems; at any level of care (health unit, neighbourhood, municipality, state, region or country).

Any primary (descriptive or analytical) or secondary study design was considered.

### Searching for studies

A broad and sensitive literature search was conducted using structured search strategies, with relevant descriptors and synonyms, for the following databases on 8 September 2021: Campbell Collaboration, Cochrane Library (via Wiley), Excerpta Medica dataBASE (Embase, via Elsevier), Biblioteca Virtual em Saúde (BVS), Epistemonikos, Health Evidence, Health Systems Evidence, Medical Literature Analysis and Retrieval System Online (MEDLINE, via PubMed) and PDQ-Evidence. A structured electronic search was conducted in the following grey literature bases on 24 February 2022: Opengrey (https://opengrey.eu), Thesis Commons (https://thesiscommons.org/) and Open Access Theses and Dissertations (https://oatd.org/).

Structured electronic searches were conducted on the following repositories of preprints on 24 February 2022: Europe PMC (https://europepmc.org/) and Open Science Preprints (https://osf.io/preprints/).

Additional unstructured searches were conducted on the following sources related to evidence-informed policy or health education on 27 February 2022: Agency for Healthcare Research and Quality AHRQ/EUA, Guidelines and Measures (www.guidelines.gov), Centre for Reviews and Dissemination (CRD), Service Delivery and Organisation (https://www.york.ac.uk/crd/research/service-delivery/), Cochrane Effective Practice and Organization of Care (EPOC) (https://epoc.cochrane.org/), EPPI-Centre (https://eppi.ioe.ac.uk/cms/Default.aspx?tabid=56), Evidence Informed Policy and Practice in Education in Europe (EIPPEE) (http://www.eippee.eu/cms/Default.aspx?tabid=3179), European Observatory on Health Systems and Policies (https://eurohealthobservatory.who.int/), ECRAN Project. European Communication on Research Awareness Needs (http://www.ecranproject.eu/en), Evidence Informed Policy Networks (EVIPNet) (https://www.who.int/initiatives/evidence-informed-policy-network), Global Evaluation Initiative (https://www.globalevaluationinitiative.org/), Informed Health Choices (https://www.informedhealthchoices.org/), International Bibliography of the Social Sciences (IBSS) (https://about.proquest.com/en/products-services/ibss-set-c/), International Initiative for Impact Evaluation (3ie) (https://www.3ieimpact.org/), McMaster University's Health Forum (https://www.mcmasterforum.org/), Rx for Change (https://www.cadth.ca/rx-change), Supporting the use of Research Evidence (SURE) (https://epoc.cochrane.org/sites/epoc.cochrane.org/files/public/uploads/SURE-Guides-v2.1/Collectedfiles/sure_guides.html, The Alliance for Health Policy and Systems Research (https://ahpsr.who.int/) and What Works Centres (https://www.gov.uk/guidance/what-works-network).

Additional unstructured searches were conducted on the following sources related to health science communication on 24 February 2022: American Medical Writers Association (AMWA, https://www.amwa.org/), European Medical Writers Association (EMWA, https://www.emwa.org/) and International Society for Medical Publication Professionals (ISMPP, https://www.ismpp.org/). A manual search was performed in reference lists of relevant studies and through contact with experts in the field.

No language filter was applied. The search was restricted to the period from the year 2000 onwards, considering the advances and changes in the digital and social media that have occurred mainly in the last two decades. Full-length publications, abstracts presented at conferences and events, online reports, theses and dissertations were included. The structured search strategies are presented in Additional file 1.

### Selecting studies

The study selection process was carried out in two phases using the Rayyan platform [16]. The first phase consisted of reading the titles and abstracts of all references retrieved by the search strategies and categorizing the studies into ‘potentially eligible’ or ‘eliminated’. The second phase consisted of reading in full the ‘potentially eligible’ studies to confirm their eligibility or exclude them in the second phase (the justifications for each exclusion in the second phase are presented). The two phases were conducted by two groups of independent researchers and inconsistencies in decisions to include or exclude studies were solved by a third researcher. The entire selection process is presented using a PRISMA flowchart.

### Extracting data

Data on the of strategies identified and included in this review were extracted by two researchers independently and inconsistencies were solved by consulting a third researcher. The following data were collected for each included study: author, year of publication, type of publication (article/report, full text/ abstract), study design, name and description of the communication strategy, institution proposing the strategy and source of funding for the study. The following data were collected, when available, for each strategy identified:

1. Strategy main category and subcategories:
2. communication of risk/benefit: including the subcategories communication of health risks and benefits under different numerical or nominal formats, health communication with positive (benefits, gains) or negative (losses) words/terms, verbal versus visual communication of the effects of interventions, communicating health risks and benefits with bar charts or bar charts and histograms, strategies for communication of health evidence and strategies for communicating risks and benefits in health with different animated graphical presentations.
3. communication of uncertainty in health: including the subcategory communication of uncertainties about the effects of interventions on health.
4. teaching/learning: including the subcategories communication/learning of key concepts related to the effects of health interventions, communication/learning resources from the IHC initiative on key concepts of evidence for health, communication/learning of key concepts of health evidence, educational podcasts from the IHC initiative on key health evidence concepts, training for parliamentarians on scientific health evidence and inclusion of stakeholders in the working group for preparing comparative effectiveness summaries.
5. evidence synthesis frameworks using plain language: including the subcategories blogshots to communicate the results of systematic reviews, evidence synthesis summary template, plain language abstract, Cochrane plain language summaries, templates for plain language abstracts of systematic reviews, printed newsletters for communicating health evidence and systematic review summaries of evidence templates for policy-makers and health system managers.
6. guidelines for elaborating/evaluating communication products: including the subcategories guidelines for designing and evaluating health evidence communication products (CDC Clear Communication Index) and tool for evaluating the quality of health texts in plain language.
7. For this categorization, a new taxonomy was elaborated with an unstructured method, which is detailed in Additional file 2.
8. Target audience: health managers, population, both.
9. Type of strategy: language, content or format of the communication.
10. Health system and level of care for which the strategy was proposed or used (public or private health, primary or specialized care; others).
11. Approach to the strategy: textual communication (printed/online material), visual communication (graphic, illustrative with drawings), verbal communication (videos, podcasts) and others.
12. Strategy length: permanent or temporary.
13. Strategy status: proposed, implemented and not evaluated, or implemented and evaluated.
14. Costs for implementing the strategy (as predicted by the authors of the studies included).
15. Barriers and facilitators for implementing the strategy (as identified by the authors of the studies included).

For the scientific evidence communication strategies that were implemented and evaluated by the included studies, information on the results was collected. These strategies were subsequently classified, at the discretion of the reviewing authors, according to the feasibility of implementation, immediate or after the adoption of actions. This classification was performed considering facilities, costs, need for regulation or local policies, and regardless of the certainty of the available evidence.

The authors of the included studies could be contacted if additional information was needed.

### Quality assessment/risk of bias of the included studies

As the aim of this scoping review is to map strategies presented in descriptive studies or to use pieces of analytical studies reporting strategies, no checklists or tools for assessing the methodological quality of the studies were applied, as recommended by the Joanna Briggs Institute for scoping reviews [13].

### Synthesis and presentation of results

Strategies were classified using the categories determined based on the data described above. A narrative synthesis was presented using graphs and/or tables. Depending on the availability of information, descriptive statistics would be performed using Microsoft Excel® and/or STATA® software, but this was not undertaken due to the format and/or scarcity of the data presented.

## Results

### Search results

Structured searches in electronic databases resulted in 25 284 references and unstructured searches in additional sources retrieved 58 references, totaling 25 342 references. After removing 744 duplicates, 24 598 references were analyzed through titles and abstracts and 24 467 were eliminated for not meeting the eligibility criteria. Thus, in the second stage of the selection process, the full texts of 131 references were analyzed. Of these, 50 were excluded [17–66] and the reasons for exclusion are detailed in Additional file 3. One reference awaits classification because, despite a series of attempts, it was not possible to obtain the full paper and the abstract did not present enough information to allow confirmation of its eligibility [67].

**Fig. 1:**
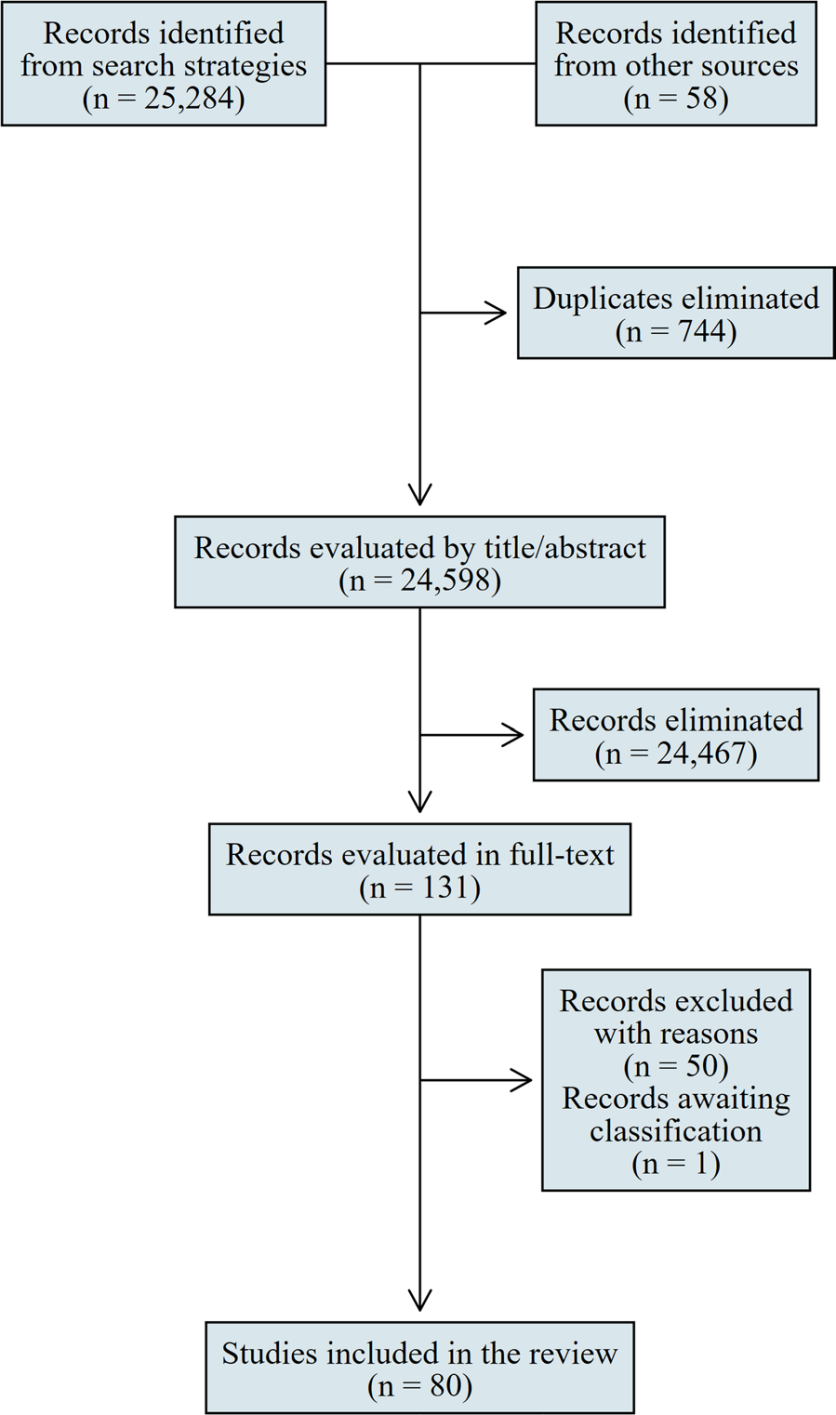
Flowchart of the study selection process

At the end of the selection process, this review included 80 studies or documents (Fig. 1) [1, 2, 6–12, 68–138].

### Characteristics of the included studies

The main characteristics of the included studies/documents are detailed in Additional file 4. Studies with a descriptive design were the most frequent (28.8%), followed by systematic reviews (16.3%) and case studies (13.8%). The studies were funded by governmental institutions or non-governmental initiatives, in the areas of health (research and assistance) and education.

### Results of included studies

Seventy-eight strategies were identified in the included studies and are presented in Additional file 5. All of the studies had the ultimate or intermediate goal of improving the comprehension of health information. None of these strategies were proposed for a specific health system or level of healthcare, and they were implemented in different scenarios (including school settings) and on a continual basis. The costs associated with the strategies were not provided by any of the studies.

Table 1 presents the main results of the strategies for communicating scientific evidence that were implemented and, to some extent, evaluated, regardless of the method used to evaluate such results (experimental study, survey, and so on).

**Table 1:** Main results of the strategies for communicating scientific evidence that were implemented and evaluated

Communication of risk/benefit on health	
Subcategory	Main results
Communication of health risks and benefits under different numerical or nominal formats	[Akl, 2011b] [7] This systematic review included 35 studies comparing the communication of health risks and benefits by natural frequency, percentages, relative risk reduction (RRR), absolute risk reduction (ARR) or number needed to treat (NNT). The main results are as follows *Natural frequencies versus percentages:* • comprehension: greater with natural frequencies than percentages (SMD 0.69; 95% CI 0.45–0.93; 642 participants; 7 comparisons; moderate-certainty evidence) *RRR versus ARR:* • comprehension: little or no difference between presentation formats (SMD 0.02; 95% CI −0.39 to 0.43; I [2] = 80%; 2 studies; moderate certainty evidence) • persuasiveness: greater with RRR (SMD 0.62; 95% CI 0.42–0.83; 15 studies; moderate-certainty evidence) *RRR versus NNT:* • comprehension: greater with RRR (SMD 0.73; 95% CI 0.43–1.04; 1 study; evidence certainty not evaluated) • persuasiveness: greater with RRR (SMD 0.65; 95% CI 0.51–0.80; 10 studies; moderate-certainty evidence) *ARR versus NNT:* • comprehension: greater with ARR (SMD 0.42; 95% CI 0.12–0.71; 1 study; moderate-certainty evidence) • persuasiveness: little or no difference between presentation formats (SMD 0.05; 95% CI −0.04 to 0.14; 10 studies; moderate-certainty evidence)
	[Büchter, 2014] [8] This systematic review included ten studies, and the main results (measured by a six-point Likert scale, suggesting small to moderate effects) were: • nominal presentation resulted in an overestimation of event risk • numerical presentation resulted in more accurate estimates, increased satisfaction with the information (MD 0.48; 95% CI 0.32–0.63; *p* < 0.00001; I [2] = 0%) and the probability of medication use
	[Chapman, 2020] [2] This overview included 44 systematic reviews on strategies for health knowledge dissemination, including strategies for communicating health risks and benefits. The main results with sufficient evidence to be implemented were: *Natural frequencies versus percentages:* • comprehension (about outcomes of health intervention effects): greater with the use of natural frequencies than percentages *RRR versus ARR:* • comprehension: no difference between the formats • persuasiveness: higher with RRR *RRR versus NNT:* • comprehension: higher with RRR • persuasiveness: higher with RRR A*RR versus NNT:* • comprehension: higher with ARR • persuasiveness: little or no difference between the formats *Numerical versus nominal (textual, printed) communication:* • satisfaction: for reporting adverse event risk in printed materials, satisfaction was significantly higher with numerical communication
	[Fortin, 2001] [85] This survey collected the opinion of 15 women about different ways of presenting risks related to hormone replacement therapy. The main results were: • 83% of the participants preferred bar to line graphs, survival curves and visual scales with facial expressions; • mortality estimates were preferred over 10 or 20 year survival • there was a preference for absolute risks over relative risks and NNT
	[Ghosh, 2005] [88] This narrative review presented the following results about strategies for communication of risk for the population: • there was a preference for ARR over NNT • the ability to interpret graphs was limited • for the population aged 75 years and over, there was a preference for graphs over percentages
	[Knapp, 2004] [96] This randomized controlled trial (RCT) included 120 participants taking simvastatin or atorvastatin after cardiac surgery or heart attack: 60 received a text communicating the risk of adverse events (constipation or pancreatitis) in nominal format, and 60 received the same text but with numerical reporting of the risks (for constipation, ‘common’ or 2.5%; for pancreatitis, ‘rare’ or 0.04%). The main results were: • estimated mean probability of constipation: 34.2% in the nominal communication group and 8.1% in the numerical communication group; for pancreatitis: 18% in the nominal communication group and 2.1% in the numerical communication group • nominal communication was associated with more negative perceptions of medications than equivalent numerical communication • nominal risk communication overrides the harm level and could lead patients to make inappropriate decisions about whether to use the medication
	[Kristiansen, 2012] [98] This survey interviewed participants (general population) who were communicated about the risk of heart attack with the use of a hypothetical medication using the NNT. The results showed that: • the NNT did not communicate information about the proportion of patients who benefited from an intervention or the extent to which an adverse event was being prevented • 80% of participants agreed with the benefit of the medication, regardless of NNT • some people who disagreed with the benefit of the medication misinterpreted the NNT • the population may have difficulty understanding the meaning of NNT, and its use should be avoided for this target audience
	[Lipkus, 2007] [100] The main results of this narrative review are presented below: *Numerical risk communication:* • people generally preferred numerical information over other formats (for example, nominal categories for probability: unlikely or very likely) • among numerical formats, natural frequencies were easier to understand • being consistent with using numerical formats, for example, not comparing percentages with probabilities or frequencies, facilitated understanding • using the same numerical denominator (for example, comparing 5 out of 100 with 15 out of 100) facilitated comparisons and reduced cognitive effort • in general, individuals more easily understood base-10 denominators (for example, 10, 100, 1000) • rounding numbers and avoiding decimals made it easier to comprehend (for example, it was easier to comprehend 30 than 29.6) • expressing a ratio as small numbers (for example, 1 in 10) led to fewer perceptions of the probability of events than the same ratio incorporating larger numbers (for example, 10 in 100) • specifying the relative risk and including the absolute risk were more comprehensive (for example, the risk of non-smokers getting the disease is 1%, while the risk of smokers is 10%, so smokers have a ten times higher risk of getting the disease than non-smokers) *Nominal risk communication:* • when nominal risk communication was the chosen format, using the main term and its variations added some objectivity and allowed comparisons (for example: likely, unlikely, very likely) *Visual risk communication:* • bar charts (histograms) were best suited for making comparisons, especially for subgroups (for example, comparing the magnitude of risk by ethnic group or sex) • line graphs (survival curves) were best suited to show trends over time and perhaps interactions between risk factors
	[McCormack, 2013] [1] This systematic review included 61 studies about communication strategies. The main results are presented below *Communication about benefits – effectiveness:* • people who received non-numerical or factual communication about medication with a higher probability of benefit for myocardial infarction chose this medication more often than people who did not receive this communication (one study, low strength of evidence) • receiving additional non-numerical information about benefits had little effect on refusals of cancer screening tests, but receiving non-numerical information about harms significantly increased refusals to screening tests and significantly decreased satisfaction with the decision (one study, low strength of evidence) *Communication about precision/imprecision:* • risk communication with numerical point estimates versus reporting with 95% CI: studies showed varying results depending on the outcome, the range of the 95% CI and the presence or absence of comparative information on the population mean risk • numerical versus graphical communication of 95% CI regarding risk perception: uncertainty evidence (one study, insufficient strength of evidence) *Communication about direct evidence:* • choosing a cholesterol medication for which there was direct evidence of benefit was more frequent among people who received non-numerical communication or factual information with direct evidence encouraging the choice of the medication than those who did not receive this communication (one study, insufficient strength of evidence)
	[Sheridan, 2003] [128] This RCT included 407 participants who were randomized to receive one of four formats of risk communication about the comparative effects of two medications: RRR (*n* = 97), ARR (*n* = 108), NNT (*n* = 100) or a combination of all three formats (*n* = 98). The main results were: • comprehension of the comparative effectiveness: higher in the RRR group (60% with RRR, 42% with ARR, 30% with NNT and 43% with the combination group; *p* = 0.001) • ability to calculate treatment effect from baseline disease risk: higher in the RRR group (21% with RRR, 17% with ARR, 6% with NNT and 7% with the combination; *p* = 0.004) • response on the effect calculation: 26% with RRR, 32% with ARR, 39% with NNT and 42% with the combination • greater difficulty with comparisons and estimates was observed in the subgroups of non-white, with some college education, females, persons with health problems or who had not previously discussed quantitative data with their physicians
	[Trevena, 2006] [130] This systematic review included 10 systematic reviews of RCTs and 30 additional RCTs. The main results were: • more modern communication strategies (verbal, textual, visual and electronic offered by the provider) increased patient comprehension but were more likely to do so if they were structured, adapted and/or interactive • probabilistic information was best communicated as event rates (natural frequency) rather than nominal terms (for example, much, little) and measures of effect size (such as relative risk reduction) • figures such as cartoons or graphs (for example, vertical bar graphs) aided comprehension • value clarification exercises helped in individual decision-making
Health communication with positive (benefits, gains) or negative (losses) words/terms	[Akl, 2011a] [6] This systematic review included 35 studies (16 342 participants from the general population) that compared communication of health attributes or effects of health interventions/exposures with positive (benefits, gains) or negative (losses) words/terms. The main results are below *For communicating attributes:* • comprehension (Likert scale): higher with negative words/terms; SMD −0.51; 95% CI −0.94 to −0, 22; 1 study; moderate effect size; low-certainty evidence • persuasiveness (measured as a hypothetical decision or intention or willingness to adopt an intervention, Likert scale): little or no difference with positive or negative words/terms; SMD 0.07; 95% CI −0.23 to 0.37; 11 studies; low-certainty evidence • behaviour (Likert scale): little or no difference with positive or negative words/terms; SMD 0.09; 95% CI −0.14 to 0.31; 1 study; moderate-certainty evidence *For communicating the effects of interventions/exposition:* • comprehension: no study evaluated the effect of communication as losses or gains on comprehension • persuasiveness (measured as a hypothetical decision or intention or willingness to adopt an intervention, Likert scale): for communication about treatment effects, persuasiveness was higher with words/terms signifying losses (SMD −0.50; 95% CI −1.04 to 0.04; 3 studies; moderate effect size; very low-certainty evidence) • behaviour (Likert scale): little or no difference with words/terms meaning gains or losses; SMD −0.06; 95% CI −0.15 to 0.03; 16 studies; low-certainty evidence
	[Edwards, 2001] [82] This narrative review concluded that there is a greater comprehension with the use of words/terms meaning losses versus words/terms meaning gains when communicating health risks and benefits (OR 1.18, 95% CI 1.01–1.38)
	[Gallagher, 2013] [37] This systematic review included 94 studies comparing the communication of health outcome results with words/terms meaning gains or losses. In total, 189 measures of effect size were evaluated, and the main results were: • behaviour: reporting the results of effect size measures as gains was more effective for encouraging desirable behaviour than reporting the results as losses (*p* = 0.002), particularly for skin cancer prevention, smoking cessation and physical activity
	[McCormack, 2013] [1] This systematic review included 61 studies on health communication strategies, and the main results on positive (benefits, gains) or negative terms were: • loss content communications associated with dichotomous (yes/no) narratives were more persuasive than (i) loss content communications associated with statistical information or (ii) gain content communications associated with narratives or statistical information (one study; insufficient strength of evidence)
Verbal versus visual communication of the effects of interventions	[Lopez, 2008] [102] This systematic review included five RCTs on contraceptive efficacy communication strategies. The main results were: • comprehension: higher in communication with sound slides than with the physician’s explanation (MD −19.00, 95% CI −27.52 to −10.48; one RCT) • correct answers: more frequent with efficacy communication using a category table compared to a numerical table (OR 2.42, 95% CI 1.43–4.12) and when compared to a category/numeric table (OR 2.58. 95% CI 1.5–4.42; one RCT)
Communicating health risks and benefits with bar charts or bar charts and histograms	[Ghosh, 2008] [89] This RCT included 150 women: 74 were randomized to receive communication via bar charts (categoric bars) and 76 via bar charts and histograms (frequency diagrams, bars with a range of values). The main results were: • 72% of the women overestimated their risk of breast cancer before the interventions • the frequency of women who had improved comprehension of this risk was not different between the different communication strategies (42% versus 54%; *p* = 0.1) • for the subgroup that overestimated the risk before the interventions, improvements in the estimate accuracy were more frequent in those receiving communication with bar charts and frequency diagrams (19% versus> 9%, *p* = 0.004)
Strategies for communication of health evidence	[Epstein, 2004] [83] This systematic review included eight studies, and the main results were: • the order of communicating the information and the outcomes may distort the comprehension of the population • when evidence was limited, using graphs or figures with human faces representing probabilities and vertical bar charts for comparative information were helpful • less-educated and older people preferred proportions to percentages and did not comprehend confidence intervals • the absolute risk was better comprehended than RRR • review authors suggested five aspects that should be considered when communicating scientific evidence to the population: comprehension of the experience and expectations of the patient (and their family members), building partnerships, providing evidence (including a balanced discussion of uncertainties), presenting recommendations informed by clinical judgment and patient preferences, and checking for comprehension and agreement
	[Grimshaw, 2012] [11] This narrative review presented some strategies for communicating health evidence divided into three groups *Decision support strategies (to assist choices about health treatment options):* • when compared with no strategy, decision support improved knowledge and risk accuracy perceptions, reduced the proportion of people who were passive in decision-making, resulted in a higher proportion of patients reaching informed decisions consistent with their values, reduced the number of people who remained undecided, reduced decision-making conflict and reduced choice for elective major surgery options favouring conservative options. Decision support did not impact satisfaction; however, further research is needed to clarify its effects on adherence to the chosen option, patient–professional communication, its cost-effectiveness and its impact on low-literate or developing populations (86 RCTs, 20 209 participants) *Personalized risk communication (information focusing on a personal interest using, for example, epidemiological calculation methods for risk calculations):* • personalized risk communication (textual, verbal or visual): increased uptake of screening tests for health conditions (weak evidence, consistent with a small effect; 22 RCT *Communication before consultations (any intervention delivered before a medical visit to help the patient to clarify his/her doubts during consultations):* • compared with control, communication before consultations increased questions asked during these consultations. Both verbal counselling and textual intervention produced similar effects on questions, but counselling increased patient satisfaction (33 RCT, 8244 participants)
Strategies for communicating risks and benefits in health with different animated graphical presentations	[Zikmund-Fisher, 2012] [137] This RCT included 4198 participants who were randomized to receive risk–benefit outcomes of health interventions based on ten different graphical presentations. Following this, participants were asked to choose the most effective and safest treatment. The probability of the participant choosing the ‘correct’ treatment with each type of graph was: • static grouped: OR 1 • static scattered: OR 0.59; 95% CI 0.38–0.91 • scatter, settles: OR 0.67; 95% CI 0.43–1.03 • grouped, built: OR 1.01; 95% CI 0.64–1.60 • scatter, built: OR 0.75; 95% CI 0.48–1.18 • scatter, built, settles: OR 0.80; 95% CI 0.51–1.26 • scatter, auto shuffles: OR 0.64; 95% CI 0.40–1.00 • scatter, auto shuffles, settles: OR 0.94; 95% CI 0.59–1.49 • scatter, user shuffles: OR 0.52; 95% CI 0.34–0.80 • scatter, user shuffles, settles: OR 0.81; 95% CI 0.52–1.27 After interpreting the results, the authors concluded that the proposed animated risk graphics showed no benefits over the traditional charts. In some cases, the animated graphs worsened the communication of risks and benefits of the intervention

Communication of uncertainty in health	
Subcategory	Main results
Communication of uncertainties about the effects of interventions on health	[Büchter, 2020] [73] In this RCT, eight versions of a summary about the effects of medication for tinnitus were compared. The versions varied in degree, type and magnitude (number of reasons) of uncertainty. Overall, 1727 participants were randomized to receive one of these versions, and the following results were reported: • perception of treatment efficacy: no difference between the methods of presenting the degree and type of uncertainty; as to the method of presenting the magnitude of uncertainty, there was greater perception when two reasons were presented compared to three (*p* = 0.04) • certainty for judging the efficacy of treatment: no difference between the variation of showing the degree, type and magnitude of uncertainty • perception about the final body of evidence: the description of imprecision was associated with a greater perception of the limitations of the evidence than the general statement that more research is needed (*p* = 0.01) • quality of the text: no difference between the methods for presenting the degree, type and magnitude of uncertainty • the decision to use the medication: no difference between the methods for presenting the degree, type and magnitude of uncertainty

Evidence synthesis frameworks using plain language	
Subcategory	Main results
Blogshots to communicate the results of systematic reviews	[Arienti, 2018] [69] This case study reported the experience of implementing five blogshots (infographics) to communicate the results of Cochrane reviews on rehabilitation in plain language. The results of accessing each blogshot were: • yoga: 2633 views, 67 interactions, 49 access to review • vocational rehabilitation: 2697 views, 67 interactions, 23 accesses to the full review • fatigue treatment: 1712 views, 76 interactions, 39 access to review • cardiovascular rehabilitation: 3419 views, 120 interactions, 51 accesses to full review (*p* = 0.12)
Evidence synthesis summary template	[Hartling, 2018] [92] This survey collected information and opinions from managers on evidence synthesis summaries. The qualitative analysis of the results showed that: • decision-makers suggested a three-page summary with key messages, details on results, meaningful numbers/tables, and strength of evidence Detailed methods and contextual information were considered less important
Plain language abstract	[Kerwer, 2021] [95] This descriptive study evaluated, through a survey, the opinion of 166 people about 12 original (scientific) abstracts and their respective plain language abstracts reporting the results of 12 different study designs. The main results showed that the plain language versions: • presented greater readability and allowed a correct comprehension of the corresponding information • were perceived as more reliable • were able to make the reader more confident about their ability to decide based on the content learned
Cochrane plain language summaries	[Santesso, 2015] [124] This RCT included 143 participants from five countries (Canada, Norway, Argentina, Spain and Italy): 97 were randomized to receive the plain language summaries and 96 to receive the original abstract (scientific) of a Cochrane systematic review about the effects of vitamin C for the common cold. The main results were: • more participants in the plain language group comprehended the benefits and harms of treatment and the certainty of the evidence (53% versus 18%, *p* < 0.001). Comprehension occurred regardless of education level • more participants in the plain language group answered the requested questions correctly (*p* < 0.001) • reliability, accessibility, comprehensiveness and utility for helping decisions were more frequent among those who received plain language summaries
Templates for plain language abstracts of systematic reviews	[Marquez, 2018] [103] In this survey, managers evaluated two new templates and one traditional template for summaries of systematic reviews through the System Usability Scale (SUS, a score < 68 is below average usability). The SUS score (standard deviation) was 55.7 (17.2) for the traditional template, 85.5 (8.0) for the new template 1 and 86.4 (11.5) for the new template 2
Printed newsletters for communicating health evidence	[Murthy, 2012] [108] This literature review identified interrupted time series assessing the dissemination of printed newsletters based on evidence from systematic reviews. The main results were: • reduced surgery rates for prominent ear correction in children younger than 10 years (mean annual decline: −10.1%, 95% CI −7.9 to −12.3) • reduced surgery rates for prominent ear correction in children younger than 15 years (mean quarterly decline: −0.044, 95% CI −0.080 to −0.011)
Systematic review summaries of evidence templates for policymakers and health system managers	[Petkovic, 2016] [116] This systematic review included six studies assessing the use of evidence summaries by managers, and the main results showed that: • evidence summaries were more effortless to comprehend than full systematic reviews • it is unclear whether using abstracts increased the use of evidence derived from systematic reviews in decision-making • targeted and personalized messages have increased the number of evidence-based public health policies and programmes • there was little or no difference in the use of evidence summaries for decision-making regarding knowledge, comprehension or beliefs, perceived usefulness or usability • summary tables of findings with the certainty of evidence rating were considered easier to comprehend compared with full systematic reviews • reporting of study event rates and absolute differences were considered comprehensible

Guidelines for elaborating/evaluating communication products	
Subcategory	Main results
Guidelines for designing and evaluating health evidence communication products (CDC Clear Communication Index)	[Baur, 2014] [71] In this survey, 870 participants (general population) blindly assessed the quality of two versions of the same health evidence communication material: the original version and the version adjusted according to the CDC clear checklist items. Communication index for designing and evaluating health communication products (http://www.cdc.gov/healthcommunication/ClearCommunicationIndex/). The three assessed materials were: questions and answers about using thimerosal (preservative) in vaccines, a fact sheet on heart disease and a fact sheet on cell phone use and health. The results of the evaluations showed that: • on average, the original versions of the three materials scored less than 30% on the checklist, and the adjusted versions scored 90% or more • the adoption of the checklist increased the quality of health communication and evidence products for the population
Tool for evaluating the quality of health texts in plain language	[Logullo, 2019] [101] In this study, the DISCERN tool was translated, culturally adapted and had its psychometric properties evaluated in Portuguese. The tool was applied in a validation study that used the plain language summary of a Cochrane systematic review. The main results were: • the Brazilian Portuguese version had excellent internal consistency and good reproducibility • age, sex and health literacy did not interfere with the score resulting from the application of the tool

Teaching/learning	
Subcategory	Main results
Communication/learning of key concepts related to the effects of health interventions	[Cusack, 2018] [9] This systematic review included 24 studies (most at high or moderate risk of bias) on communication/learning strategies key concept effects of health interventions Strategies implemented inside and outside the school environment, single or multiple, were identified using different approaches such as discussion groups, printed material, online classes, and short- or long-term courses. The outcomes evaluated included: knowledge, skills, behaviour, confidence, perception of knowledge and/or skill, attitude and satisfaction. The main results observed were: • the effects of strategies on trust, attitude and behaviour were uncertain • improvements in the quality of studies, consistency of outcome measures, and longer-term evaluation of strategies are needed to improve reliability in estimating the effects of the strategies evaluated
Communication/learning resources from the IHC initiative on key concepts of evidence for health	[Ikirezi, 2016] [94] This case study evaluated the feasibility of implementing communication/learning resources to support the comprehension and application of key concepts in the critical assessment of the evidence in health in a preschool in Rwanda. The main results observed in the qualitative analysis were: • the user experience was positive, as implementing the IHC features was considered beneficial, contextualized, reliable, feasible and desirable • the restricted time to use the resources was considered a barrier, while curiosity and a positive attitude towards the resources were facilitators • students and faculty suggested that IHC resources be distributed to other students at other schools so they could also benefit from the teachings and importance of making evidence-informed health choices
	[Mugisha, 2016] [107] This case study reported the experience of implementing communication/learning resources to support the comprehension and application of key concepts in the critical appraisal of the evidence in health in a primary school in Rwanda. The main results observed in the qualitative analysis were: • the use of IHC resources translated into Kinyarwanda was considered viable in Rwanda • it was essential to collect suggestions and ideas from participants to contextualize the IHC resources in the local scenario • children and teachers can be helpful in evaluating and reviewing primary school resources and contribute significantly to improving educational resources that would benefit ministries of education • the resources were considered useful, feasible, reliable and comprehensible by users
	[Nsangi, 2017] [110] This cluster clinical trial included 120 schools that were randomized to receive learning resources (for example, teacher’s guides, exercise and textbooks, posters, songs and activities cards; intervention group, *n* = 60, 76 teachers and 6383 children) or not (control group, *n* = 60, 67 teachers and 4430 children). The main results were: • children's mean score on a test with two multiple-choice questions for each of the 12 key concepts in the material: 62.4% (SD 18.8) in the intervention group versus 43.1% (SD 15.2) in the control group (adjusted mean difference 20%; 95% CI 17.3–22.7; *p* < 0.00001) • the proportion of children with sufficient scores to pass the same test (≥ 13 of 24 correct answers): 69% (3967/5753) in the intervention group versus 27% (1186/4430) in the control group (adjusted difference of 50%; 95% CI 44–55) • the intervention was effective for children with different reading skills but was more effective in the subgroup of children with better reading skills
Communication/learning of key concepts of health evidence	[Nordheim, 2016] [109] This systematic review identified RCT and non-randomized studies comparing different educational strategies to acquire skills for the critical assessment of health evidence. The main results were: • active versus traditional teaching methodologies: capacity for basic knowledge about causality and association, randomization, epidemiology concepts and evidence-based medicine was 71% higher with active methods • educational strategy versus control: the ability to recognize that multiple outcomes can influence cancer research results were twice as high in the group that received an educational strategy • educational strategy with active methodology versus control: the comprehension of the need for comparative studies to make inferences about causality was 51% higher with the use of the educational strategy • fictional evidence reasoning simulation versus control group: reduction in the number of inappropriate responses, including personal beliefs and unsupported opinions, with the use of simulation
Educational podcasts from the IHC initiative on key health evidence concepts	[Ringle, 2020] [118] This RCT included 250 American parents randomized to listen to the podcasts with evidence-based health content developed by the IHC initiative (intervention group, *n* = 128) or podcasts with general information (control group, *n* = 122). The main results were: • critical thinking skill: 53% of parents in the intervention group achieved a satisfactory score on the applied skill test (> 18 and 21) versus 26.2% in the control group • satisfaction with the podcast (scale 1–5): similar between groups (4.16 ± 0.93 versus 4.20 ± 0.85) • listening to the IHC podcast improved parents’ behaviour towards evidence-based practice and preference for evidence-based health information • podcasts are available at: https://www.informedhealthchoices.org/podcast-for-parents/
	[Semakula, 2017; Semakula, 2020] [125, 126] This RCT included 675 parents of Ugandan elementary school students who were randomized to listen to podcasts with evidence-based health content (intervention group, *n* = 334) developed by the IHC initiative or podcasts with general health information used by the public service (control group, *n* = 341). The main initial and post-1 year results were: • mean critical thinking skill test score (two multiple-choice questions for each of nine key critical thinking concepts, 18 questions total): 67.8% (SD 19.6%) in the intervention group versus 52 .4% (SD 17.6%) in the control group (adjusted DM 15.5%; 95% CI 12.5–18.6%; p < 0.0001); after 1 year: 58.9% in the intervention group versus 52.6% in the control group (adjusted DM 6.7%; 95% CI 3.3–10.1%) • frequency of parents who achieved the minimum passing test score (at least 11 out of 18): 71% (203/288) in the intervention group versus 38% (103/273) in the control group (adjusted SD 34%; 95% CI 26–41%; p < 0·0001); after 1 year: 47.2% in the intervention group versus 39.5% in the control group (adjusted SD 9.8%; 95% CI 0.9–18.9%; *p* < 0.0001) • listening to the IHC initiative podcast improved parents’ ability to critically assess the information at baseline, but this ability declined substantially after 1 year
	[Semakula, 2019b] [127] This descriptive study used design thinking methodology to develop podcasts on health evidence and presented users’ assessments of the podcasts. The main results were: • usefulness: IHC podcasts were considered useful tools that could help encourage critical thinking when publicized in the general media and specific contexts (for example, schools) • usability and comprehension: were considered satisfactory • credibility: considered satisfactory • desire to use: some participants asked if they could have access to all episodes. A non-governmental health communication organization and producers from the Uganda Broadcasting Corporation expressed their interest in broadcasting the podcasts on radio as part of their health communication programming
Training for parliamentarians on scientific health evidence	[Cockcroft, 2014] [78] In this case study, the authors reported the experience of training on scientific evidence in health for 36 of Botswana’s 54 elected parliamentarians. The training took place in two sessions (one theoretical and one practical workshop). It addressed the following topics: (i) initial concepts about scientific evidence (clinical trials, randomization, statistical significance, number needed to treat and bias) and the importance of control or comparators when evaluating the effects of interventions, (ii) how biases can distort results and reports, (iii) importance of evaluating the impact on public health and not just on individual health. The short-term qualitative assessment showed that: • feedback from Botswana parliamentarians were very favourable: they requested additional sessions to address the topics in more detail, and that training be offered to other decision-makers • after the training, one of the parliamentarians reported that the debate on updating the national human immunodeficiency virus (HIV) policy was more detailed and focused on evidence
Inclusion of stakeholders in the working group for preparing comparative effectiveness summaries	[Balshem, 2011] [70] In this case study, the authors presented the process and results of including stakeholders (including managers) in preparing the summary ‘Medications to reduce the risk of primary breast cancer in women’. Stakeholders suggested that the conclusions of the summaries go beyond just saying that the evidence is insufficient and that further studies are needed. Instead, stakeholders indicated that the following issues be addressed in the summaries: • what evidence is available, and what can be learned from it? • what evidence tells us when and if an intervention is safe/harmful and effective/ineffective for relevant clinical outcomes? • what can we learn from the evidence from a study inferior to a randomized clinical trial? • what can patient records tell us about the safety of a treatment? • what evidence identifies the subpopulations most likely to benefit from its use? • what kind of evidence is needed to assess short-term and long-term effectiveness? • when is short-term evidence appropriate to be implemented?

Regarding the target audience, 71.8% of the strategies were intended for the general population, 20.5% specifically for managers and 7.7% were applicable to both groups (Additional file 5).

According to the main category, communicating risks/benefits on health represented 29.5% of the strategies and encompassed different forms, nominal (categorical) or numerical (statistical), to communicate attributes or effects of health interventions or exposures. The 17 strategies in the ‘teaching/learning’ category, comprised structural actions in schools (many of them conducted by the IHC initiative), virtual environments (websites), and even in parliament (Table 1).

According to the status, more than half (52.6%) of the identified strategies had already been implemented and evaluated in some degree, 44.9% had already been implemented but not yet evaluated and 2.6% were merely proposed without any sort of implementation or evaluation (Additional file 5).

According to the delivery approach, 88.5% of the strategy had at least one textual component, 6.4% adopted an exclusively verbal approach of communication outreach and 2.6% a exclusively visual approach (Additional file 5).

As depicted in Additional file 5, the main barriers for implementing the proposed strategies are related to stakeholder time availability [73, 92, 107, 110], high speed of publication of new studies/growing volume of information [72, 77], language [76, 99, 104, 105, 121], conflicts of interest [78] and need for continuous update [97]. The main facilitator was online free access or social media access [2, 72, 97, 104, 105].

## Discussion

This scoping review was developed to identify the evidence available on the strategies for communicating health scientific evidence to the public or managers, its characteristics and settings of implementation, as well as knowledge gaps. Overall, 80 studies, reports or other forms of information presentation were included which addressed 78 strategies. The most frequent strategies were those communicating risks and benefits in health, presenting textual delivery approach, implemented and, to some extent, evaluated. Although conclusions about effects are not the focus of a scoping review, among the strategies evaluated, those that appear to present some potential benefit are:

- Risk/benefit communication: greater comprehension with natural frequencies than with percentages; greater comprehension with absolute risk than with relative risks and NNT; greater comprehension and behaviour change with numerical communication than with nominal communication; greater comprehension of mortality than of survival; communications with negative or loss content appear to be more useful for comprehension, satisfaction, and behaviour change than communications with positive or gain content; nominal communication can lead to overestimation of the risk of adverse events and can lead patients to make inappropriate decisions about whether or not to use a medication.
- Evidence synthesis templates and other plain language documents: plain language summaries to communicate the results of Cochrane systematic reviews to the population were perceived to be more reliable, easier to find and understand, and better to support decisions than the original summaries.
- Teaching/learning: the IHC initiative’s resources for communication and learning of key health evidence concepts appear to be effective in improving critical thinking skills in health immediately after their use; however, these effects were not observed after 1 year; theoretical–practical training for parliamentarians on scientific evidence in health seems to be a strategy with potential to raise awareness and improve the comprehension of this subgroup of managers on health-related evidence.

The main strengths of this scoping review involve a broad (across multiple sources of information) and sensitive (search strategies including also synonyms and free terms) search. As shown in Fig. 1, 24 598 references were screened in the first phase by reading titles and abstracts. Other features that endow methodological robustness are: the availability of a prospectively developed protocol, the selection and extraction of data in a duplicative and independent mode, and the adoption of methods recommended by the Joanna Briggs Institute Manual for scoping reviews [13].

One strength was the identification of communication strategies that used structuring learning approaches to continuously and progressively build a more favourable scenario for the population and managers to receive communication products. In this respect, the IHC initiative (https://www.informedhealthchoices.org/) and the ECRAN project (http://ecranproject.eu/) were particularly noteworthy.

For the categorization of communication strategies, different taxonomies have been identified in the literature that could be somewhat adapted for use in this scoping review [1, 99, 139, 140]. These taxonomies covered health communication in a broad sense, including mainly guidance on diagnostic, prophylactic and therapeutic conducts, many of them focusing on the individual and on the professional–patient relationship. Others had as the target audience mainly managers and health professionals, while others involved the whole process of knowledge translation and/or evidence implementation.

The particularities of health communication strategies with a specific focus on scientific evidence difficult reproducible and consistent adaptations from these aforementioned taxonomic tools. Thus, while conducting this scoping review, the authors developed, by means of an unstructured method, a proposal for a particular taxonomy for this scenario (Additional file 2). Although innovative, and having fulfilled its role within this scoping review, the proposed taxonomy has been applied for the first time and has not been formally evaluated, so limitations may be identified throughout its use hereafter.

When planning this review (protocol phase), there was no nominal definition of possible strategies. Along the construction of the search strategies, the term ‘risk communication’ and its synonyms were not used, but instead, less specific terms were used to sensitize the search. However, throughout the study selection process, a considerable number of studies specific to risk communication were identified. Thus, although 29.9% of the communication strategy included were specific to risk communication and health benefits, it is not possible to rule out that studies targeting this approach were not retrieved.

Another concern was that despite a number of different attempts (including messages to the authors, contacting experts and searching international libraries) it was not possible to obtain the full text of one of the identified studies [67]. The reading of the abstract did not allow us to confirm or refute the adequacy to the eligibility criteria and therefore this study remained as ‘awaiting classification’.

Some studies addressed combined strategies and it was not possible to quantify the exact number of different strategies addressed in the 78 strategies identified given a high rate of overlap of their components. To mitigate this shortcoming, we have chosen to present a detailed (and therefore longer than we would have preferred) table describing each strategy (Additional file 5).

Although 52.6% of the identified strategy were implemented and evaluated, much of these evaluations were characterized by opinions and satisfaction surveys. Few were evaluated through comparative studies capable of estimating their efficacy with more certainty and less bias. Additionally, the studies showed that most of the outcomes evaluated were limited to assessing comprehension, persuasion and customer satisfaction; few studies assessed health behaviour change, and none considered clinical outcomes.

Part of the strategies categorized as teaching/learning have been implemented and evaluated in African countries such as Rwanda [94], Uganda [110, 125, 126] and Botswana [78] which allowed us to evaluate the impact of these strategies, customer experience, barriers and facilitators in settings with limited financial and social resources.

As implications for practice, the identification of communication strategies that have been implemented and evaluated (Table 1) can support social, academic, governmental or non-governmental actions. Considering aspects such as feasibility, costs, need for regulation or local policies, regardless of the certainty of the available evidence, this review identified strategies that have already been evaluated in some way and that are potentially implementable in resource-scarce settings.

Strategies for communicating health risks and benefits, including attribute results and effects of interventions and exposures on health outcomes, were evaluated by studies with appropriate designs, with reliable results that could be implemented. An example is the benefit of communication using absolute frequencies and standardized decimal denominators (20 people out of 100 people using this drug might get diarrhea) rather than relative risk or NNT.

The elaboration and dissemination of communication products in parallel with scientific publications, and aimed at different audiences, is a reality (as exemplified by Cochrane’s plain language summary). This approach could be replicated and adopted by other organizations or scientific publishers, using results from reviews such as this one. In the same direction, the Brazilian Ministry of Health, in a recent initiative in partnership with the *Escola Nacional de Administração* (ENAP, National School of Administration), is producing prototype products for communicating scientific evidence in accessible language using design thinking methodology [141, 142].

As implications for forthcoming research, this scoping review identified a number of knowledge gaps that still need to be addressed by studies with appropriate designs and methods. These gaps include evidence on (i) the efficacy of communication strategies on outcome measures, such as behaviour change and clinical benefits related to the control or prevention of health conditions, (ii) the costs associated with implementing the strategies, (iii) effects of the strategies for low-income, lower sociocultural and/or resource-poor populations, and (iv) effects of the strategies for subgroups such as the elderly, adolescents and children.

## Conclusions

This scoping review identified 80 studies, reports or other documents that addressed 78 strategies for communicating scientific health evidence to the population and/or managers. Some of these strategies have been implemented and evaluated, and may have some benefit in improving these audiences’ comprehension of evidence concepts and promoting behaviour change. The findings of this review have important potential for applicability in the area of evidence-informed policy, with direct application or adaptation of identified strategies to improve the communication of scientific evidence on healthcare to managers and the population. Future efforts are needed to evaluate the effects of evidence communication strategies on relevant clinical outcomes, identify the most appropriate strategies for different settings and contexts, and promote the use of those strategies that show benefits for individual or public health and health systems.

## Supplementary Information

**Additional file 1.** Strategies of electronic and structured searches.

**Additional file 2.** Proposed taxonomy to categorize strategies for communicating scientific evidence in health to the population/managers.

**Additional file 3.** Studies/documents excluded and reasons for exclusions after reading the full text (second phase of the selection process).

**Additional file 4.** Main characteristics of included studies.

**Additional file 5.** Main characteristics of the identified strategies or sets of strategies for communicating scientific evidence.

## Data Availability

All relevant data is presented as supplementary files.
