# Supplementary material for: Strategies for communicating scientific evidence on healthcare to managers and the population: a scoping review
Source: Health Res Policy Syst. 2023 Jul 10;21:71. doi: 10.1186/s12961-023-01017-2 (PMC10334604; doi:10.1186/s12961-023-01017-2)
Supplement: Supplementary file 1 — Additional file 1. Strategies of electronic and structured searches. [file 12961_2023_1017_MOESM1_ESM.docx]

**Additional Material 1**. Strategies of electronic and structured searches.

| **Databases or repositories** | | |
| --- | --- | --- |
| **Source** | **Search strategy** | **Results (08/09/2021)** |
| Campbell Collaboration | #1 "Health Communication" OR "Communication, Health" OR "Communications, Health" OR "Health Communications" OR "Translational Medical Research" OR "Medical Research, Translational" OR "Research, Translational Medical" OR "Translational Medical Science" OR "Medical Science, Translational" OR "Medical Sciences, Translational" OR "Science, Translational Medical" OR "Sciences, Translational Medical" OR "Translational Medical Sciences" OR "Translational Research, Medical" OR "Medical Translational Research" OR "Research, Medical Translational" OR "Translational Medicine" OR "Medicine, Translational" OR "Knowledge Translation" OR "Knowledge Translations" OR "Translation, Knowledge" OR "Translations, Knowledge" OR "Translational Research" OR "Research, Translational" OR "Translational Researchs" | 2 |
| Cochrane Library  (via Wiley) | #1 MeSH descriptor: [Health Communication] explode all trees  #2 "Communication, Health" OR "Communications, Health" OR "Health Communications"  #3 MeSH descriptor: [Translational Medical Research] explode all trees  #4 "Translational Medical Research" OR "Medical Research, Translational" OR "Research, Translational Medical" OR "Translational Medical Science" OR "Medical Science, Translational" OR "Medical Sciences, Translational" OR "Science, Translational Medical" OR "Sciences, Translational Medical" OR "Translational Medical Sciences" OR "Translational Research, Medical" OR "Medical Translational Research" OR "Research, Medical Translational" OR "Translational Medicine" OR "Medicine, Translational" OR "Knowledge Translation" OR "Knowledge Translations" OR "Translation, Knowledge" OR "Translations, Knowledge" OR "Translational Research" OR "Research, Translational" OR "Translational Researchs"  #5 #1 OR #2 OR #3 OR #4  #6 MeSH descriptor: [Evidence-Based Practice] explode all trees  #7 "Evidence-Based Practice" OR "Evidence Based Practice" OR "Evidence Based Management, Healthcare" OR "Evidence Based Health Care Management" OR "Evidence Based Healthcare Management" OR "Evidence Based Management, Health Care" OR "Evidence-Based Health Care" OR "Evidence Based Health Care" OR "Evidence-Based Health Cares" OR "Health Care, Evidence-Based" OR "Health Cares, Evidence-Based" OR "Evidence-Based Healthcare" OR "Evidence Based Healthcare" OR "Evidence-Based Healthcares" OR "Healthcare, Evidence-Based" OR "Healthcares, Evidence-Based" OR "EBP" OR "Evidence-based information" OR "Evidence-based policy" OR "Health-related evidence"  #8 #6 OR #7  #9 #5 AND #8 with Cochrane Library publication date from Jan 2000 to Sep 2021, in Cochrane Reviews and Trials | 100 |
| EMBASE  (via Elsevier) | #1 'medical information'/exp OR 'communication, health' OR 'communications, health' OR 'health communications'  #2 'knowledge translation'/exp OR 'Translational Medical Research' OR 'Medical Research, Translational' OR 'Research, Translational Medical' OR 'Translational Medical Science' OR 'Medical Science, Translational' OR 'Medical Sciences, Translational' OR 'Science, Translational Medical' OR 'Sciences, Translational Medical' OR 'Translational Medical Sciences' OR 'Translational Research, Medical' OR 'Medical Translational Research' OR 'Research, Medical Translational' OR 'Translational Medicine' OR 'Medicine, Translational' OR 'Knowledge Translation' OR 'Knowledge Translations' OR 'Translation, Knowledge' OR 'Translations, Knowledge' OR 'Translational Research' OR 'Research, Translational' OR 'Translational Researchs'  #3 #1 AND #2  #4 'evidence based practice'/exp OR 'Evidence Based Practice' OR 'Evidence Based Management, Healthcare' OR 'Evidence Based Health Care Management' OR 'Evidence Based Healthcare Management' OR 'Evidence Based Management, Health Care' OR 'Evidence-Based Health Care' OR 'Evidence Based Health Care' OR 'Evidence-Based Health Cares' OR 'Health Care, Evidence-Based' OR 'Health Cares, Evidence-Based' OR 'Evidence-Based Healthcare' OR 'Evidence Based Healthcare' OR 'Evidence-Based Healthcares' OR 'Healthcare, Evidence-Based' OR 'Healthcares, Evidence-Based' OR 'EBP' OR 'Evidence-based information' OR 'Evidence-based policy' OR 'Health-related evidence'  #5 #3 AND #4  #6 #5 AND [embase]/lim NOT ([embase]/lim AND [medline]/lim) AND (2000:py OR 2001:py OR 2002:py OR 2003:py OR 2004:py OR 2005:py OR 2006:py OR 2007:py OR 2008:py OR 2009:py OR 2010:py OR 2011:py OR 2012:py OR 2013:py OR 2014:py OR 2015:py OR 2016:py OR 2017:py OR 2018:py OR 2019:py OR 2020:py OR 2021:py) | 7.904 |
| Biblioteca Virtual em Saúde (BVS) | #1 MH:(Comunicação em Saúde) OR (Health Communication) OR (Comunicación en Salud) OR L01.143.350 OR N02.208 OR SP2.021.167 OR (Communication, Health) OR (Communications, Health) OR (Health Communications) OR MH:(Pesquisa Médica Translacional) OR (Translational Medical Research) OR (Investigación en Medicina Traslacional) OR H01.770.644.145.675 OR (Medical Research, Translational) OR (Research, Translational Medical) OR (Translational Medical Science) OR (Medical Science, Translational) OR (Medical Sciences, Translational) OR (Science, Translational Medical) OR (Sciences, Translational Medical) OR (Translational Medical Sciences) OR (Translational Research, Medical) OR (Medical Translational Research) OR (Research, Medical Translational) OR (Translational Medicine) OR (Medicine, Translational) OR (Knowledge Translation) OR (Knowledge Translations) OR (Translation, Knowledge) OR (Translations, Knowledge) OR (Translational Research) OR (Research, Translational) OR (Translational Researchs)  #2 MH:(Prática Clínica Baseada em Evidências) OR (Evidence-Based Practice) OR (Práctica Clínica Basada en la Evidencia) OR H02.249 OR (Evidence Based Practice) OR (Evidence Based Management, Healthcare) OR (Evidence Based Health Care Management) OR (Evidence Based Healthcare Management) OR (Evidence Based Management, Health Care) OR (Evidence-Based Health Care) OR (Evidence Based Health Care) OR (Evidence-Based Health Cares) OR (Health Care, Evidence-Based) OR (Health Cares, Evidence-Based) OR (Evidence-Based Healthcare) OR (Evidence Based Healthcare) OR (Evidence-Based Healthcares) OR (Healthcare, Evidence-Based) OR (Healthcares, Evidence-Based) OR (EBP) OR (Evidence-based information) OR (Evidence-based policy) OR (Health-related evidence)  #3 #1 AND #2 | 536 |
| Epistemonikos | #1 (title:((Health Communication) OR (Communication, Health) OR (Communications, Health) OR (Health Communications)) OR abstract:((Health Communication) OR (Communication, Health) OR (Communications, Health) OR (Health Communications) OR (Medical Research, Translational) OR (Research, Translational Medical) OR (Translational Medical Science) OR (Medical Science, Translational) OR (Medical Sciences, Translational) OR (Science, Translational Medical) OR (Sciences, Translational Medical) OR (Translational Medical Sciences) OR (Translational Research, Medical) OR (Medical Translational Research) OR (Research, Medical Translational) OR (Translational Medicine) OR (Medicine, Translational) OR (Knowledge Translation) OR (Knowledge Translations) OR (Translation, Knowledge) OR (Translations, Knowledge) OR (Translational Research) OR (Research, Translational) OR (Translational Researchs))  #2 (title:((Evidence-Based Practice) OR (Evidence Based Practice) OR (Evidence Based Management, Healthcare) OR (Evidence Based Health Care Management) OR (Evidence Based Healthcare Management) OR (Evidence Based Management, Health Care) OR (Evidence-Based Health Care) OR (Evidence Based Health Care) OR (Evidence-Based Health Cares) OR (Health Care, Evidence-Based) OR (Health Cares, Evidence-Based) OR (Evidence-Based Healthcare) OR (Evidence Based Healthcare) OR (Evidence-Based Healthcares) OR (Healthcare, Evidence-Based) OR (Healthcares, Evidence-Based) OR "EBP" OR Evidence OR (Evidence-based information) OR (Evidence-based policy) OR (Health-related evidence)) OR abstract:((Evidence-Based Practice) OR (Evidence Based Practice) OR (Evidence Based Management, Healthcare) OR (Evidence Based Health Care Management) OR (Evidence Based Healthcare Management) OR (Evidence Based Management, Health Care) OR (Evidence-Based Health Care) OR (Evidence Based Health Care) OR (Evidence-Based Health Cares) OR (Health Care, Evidence-Based) OR (Health Cares, Evidence-Based) OR (Evidence-Based Healthcare) OR (Evidence Based Healthcare) OR (Evidence-Based Healthcares) OR (Healthcare, Evidence-Based) OR (Healthcares, Evidence-Based) OR (EBP) OR (Evidence-based information) OR (Evidence-based policy) OR (Health-related evidence)))  #3 #1 AND #2 (Custom year range: 2000 to 2021) | 43 |
| Health Evidence | [("Health Communication" OR "Communication, Health" OR "Communications, Health" OR "Health Communications" OR "Translational Medical Research" OR "Medical Research, Translational" OR "Research, Translational Medical" OR "Translational Medical Science" OR "Medical Science, Translational" OR "Medical Sciences, Translational" OR "Science, Translational Medical" OR "Sciences, Translational Medical" OR "Translational Medical Sciences" OR "Translational Research, Medical" OR "Medical Translational Research" OR "Research, Medical Translational" OR "Translational Medicine" OR "Medicine, Translational" OR "Knowledge Translation" OR "Knowledge Translations" OR "Translation, Knowledge" OR "Translations, Knowledge" OR "Translational Research" OR "Research, Translational" OR "Translational Researchs") AND ("Evidence-Based Practice" OR "Evidence Based Practice" OR "Evidence Based Management, Healthcare" OR "Evidence Based Health Care Management" OR "Evidence Based Healthcare Management" OR "Evidence Based Management, Health Care" OR "Evidence-Based Health Care" OR "Evidence Based Health Care" OR "Evidence-Based Health Cares" OR "Health Care, Evidence-Based" OR "Health Cares, Evidence-Based" OR "Evidence-Based Healthcare" OR "Evidence Based Healthcare" OR "Evidence-Based Healthcares" OR "Healthcare, Evidence-Based" OR "Healthcares, Evidence-Based" OR "EBP" OR Evidence OR "Evidence-based information" OR "Evidence-based policy" OR "Health-related evidence" OR abstract:""Evidence-Based Practice" OR "Evidence Based Practice" OR "Evidence Based Management, Healthcare" OR "Evidence Based Health Care Management" OR "Evidence Based Healthcare Management" OR "Evidence Based Management, Health Care" OR "Evidence-Based Health Care" OR "Evidence Based Health Care" OR "Evidence-Based Health Cares" OR "Health Care, Evidence-Based" OR "Health Cares, Evidence-Based" OR "Evidence-Based Healthcare" OR "Evidence Based Healthcare" OR "Evidence-Based Healthcares" OR "Healthcare, Evidence-Based" OR "Healthcares, Evidence-Based" OR "EBP" OR "Evidence-based information" OR "Evidence-based policy" OR "Health-related evidence")] AND Limit: Date = Published from 2000 to 2021 | 49 |
| Health Systems Evidence | ((Health Communication) OR (Communication, Health) OR (Communications, Health) OR (Health Communications) OR (Translational Medical Research) OR (Medical Research, Translational) OR (Research, Translational Medical) OR (Translational Medical Science) OR (Medical Science, Translational) OR (Medical Sciences, Translational) OR (Science, Translational Medical) OR (Sciences, Translational Medical) OR (Translational Medical Sciences) OR (Translational Research, Medical) OR (Medical Translational Research) OR (Research, Medical Translational) OR (Translational Medicine) OR (Medicine, Translational) OR (Knowledge Translation) OR (Knowledge Translations) OR (Translation, Knowledge) OR (Translations, Knowledge) OR (Translational Research) OR (Research, Translational) OR (Translational Researchs)) AND ((Evidence-Based Practice) OR (Evidence Based Practice) OR (Evidence Based Management, Healthcare) OR (Evidence Based Health Care Management) OR (Evidence Based Healthcare Management) OR (Evidence Based Management, Health Care) OR (Evidence-Based Health Care) OR (Evidence Based Health Care) OR (Evidence-Based Health Cares) OR (Health Care, Evidence-Based) OR (Health Cares, Evidence-Based) OR (Evidence-Based Healthcare) OR (Evidence Based Healthcare) OR (Evidence-Based Healthcares) OR (Healthcare, Evidence-Based) OR (Healthcares, Evidence-Based) OR (EBP) OR Evidence OR (Evidence-based information) OR (Evidence-based policy) OR (Health-related evidence)) | 0 |
| MEDLINE  (via PubMed) | #1 "Health Communication"[Mesh] OR (Communication, Health) OR (Communications, Health) OR (Health Communications)  #2 "Translational Medical Research"[Mesh] OR (Medical Research, Translational) OR (Research, Translational Medical) OR (Translational Medical Science) OR (Medical Science, Translational) OR (Medical Sciences, Translational) OR (Science, Translational Medical) OR (Sciences, Translational Medical) OR (Translational Medical Sciences) OR (Translational Research, Medical) OR (Medical Translational Research) OR (Research, Medical Translational) OR (Translational Medicine) OR (Medicine, Translational) OR (Knowledge Translation) OR (Knowledge Translations) OR (Translation, Knowledge) OR (Translations, Knowledge) OR (Translational Research) OR (Research, Translational) OR (Translational Researchs)  #3 #1 OR #2  #4 "Evidence-Based Practice"[Mesh] OR (Evidence Based Practice) OR (Evidence Based Management, Healthcare) OR (Evidence Based Health Care Management) OR (Evidence Based Healthcare Management) OR (Evidence Based Management, Health Care) OR (Evidence-Based Health Care) OR (Evidence Based Health Care) OR (Evidence-Based Health Cares) OR (Health Care, Evidence-Based) OR (Health Cares, Evidence-Based) OR (Evidence-Based Healthcare) OR (Evidence Based Healthcare) OR (Evidence-Based Healthcares) OR (Healthcare, Evidence-Based) OR (Healthcares, Evidence-Based) OR (EBP) OR (Evidence-based information) OR (Evidence-based policy) OR (Health-related evidence)  #5 #3 AND #4  Filters applied: from 2000/1/1 - 2021/8/19. | 16.446 |
| PDQ-Evidence | #1 (title:((Health Communication) OR (Communication, Health) OR (Communications, Health) OR (Health Communications) OR (Research, Translational Medical) OR (Translational Medical Science) OR (Medical Science, Translational) OR (Medical Sciences, Translational) OR (Science, Translational Medical) OR (Sciences, Translational Medical) OR (Translational Medical Sciences) OR (Translational Research, Medical) OR (Medical Translational Research) OR (Research, Medical Translational) OR (Translational Medicine) OR (Medicine, Translational) OR (Knowledge Translation) OR (Knowledge Translations) OR (Translation, Knowledge) OR (Translations, Knowledge) OR (Translational Research) OR (Research, Translational) OR (Translational Researchs)))  #2 (title:((Evidence-Based Practice) OR (Evidence Based Practice) OR (Evidence Based Management, Healthcare) OR (Evidence Based Health Care Management) OR (Evidence Based Healthcare Management) OR (Evidence Based Management, Health Care) OR (Evidence-Based Health Care) OR (Evidence Based Health Care) OR (Evidence-Based Health Cares) OR (Health Care, Evidence-Based) OR (Health Cares, Evidence-Based) OR (Evidence-Based Healthcare) OR (Evidence Based Healthcare) OR (Evidence-Based Healthcares) OR (Healthcare, Evidence-Based) OR (Healthcares, Evidence-Based) OR "EBP" OR Evidence OR (Evidence-based information) OR (Evidence-based policy) OR (Health-related evidence)) OR abstract:((Evidence-Based Practice) OR (Evidence Based Practice) OR (Evidence Based Management, Healthcare) OR (Evidence Based Health Care Management) OR (Evidence Based Healthcare Management) OR (Evidence Based Management, Health Care) OR (Evidence-Based Health Care) OR (Evidence Based Health Care) OR (Evidence-Based Health Cares) OR (Health Care, Evidence-Based) OR (Health Cares, Evidence-Based) OR (Evidence-Based Healthcare) OR (Evidence Based Healthcare) OR (Evidence-Based Healthcares) OR (Healthcare, Evidence-Based) OR (Healthcares, Evidence-Based) OR "EBP" OR (Evidence-based information) OR (Evidence-based policy) OR (Health-related evidence)))  #3 #1 AND #2 (Custom year range: 2000 to 2021) | 56 |
| **Grey literature** | | |
| **Source** | **Search strategy** | **Results**  **24/02/22** |
| Opengrey | ("Knowledge Translation" OR “Health Communication") AND (Evidence-Based Practice” OR “Evidence-Based Medicine”) | 0 |
| Thesis Commons | (Knowledge Translation OR Translational Medical Research OR Health Communication) AND Evidence-Based Medicine  Filters (by subject): Medicine and Health Sciences; Life Sciences; Social and Behavioral Sciences. | 12 |
| Open Access Theses and Dissertations | "Health Communication" AND "Evidence-based Medicine" OR "Evidence-based Health". | 3 |
| **Preprint repositories** | | |
| **Source** | **Search strategy** | **Results**  **24/02/22** |
| Europe PMC | ("Knowledge Translation" OR “Health Communication") AND (Evidence-Based Practice” OR “Evidence-Based Medicine”) | 28 |
| Open Science Preprints | "Health Communication" "Evidence-based medicine" "Knowledge Translation"  Filter by subject: Education; Life Sciences; Medicine and Health Sciences; Social and Behavioral Sciences. | 105 |
| Total | **-----** | **25.284** |

*BVS: Biblioteca Virtual em Saúde; LILACS: MEDLINE: Medical Literature Analysis and Retrieval System Online; PMC: PubMed Central*
