# Supplementary material for: Strategies for communicating scientific evidence on healthcare to managers and the population: a scoping review
Source: Health Res Policy Syst. 2023 Jul 10;21:71. doi: 10.1186/s12961-023-01017-2 (PMC10334604; doi:10.1186/s12961-023-01017-2)
Supplement: Supplementary file 2 — Additional file 2. Proposed taxonomy to categorize strategies for communicating scientific evidence in health to the population/managers. [file 12961_2023_1017_MOESM2_ESM.docx]

**Additional Material 2**. Proposed taxonomy to categorize strategies for communicating scientific evidence in health to the population/managers.

| **Category** | **Description** |
| --- | --- |
| **Communication of risk/benefit in health,** | Strategies, encompassing different languages and formats, to communicate risks, risk reduction and other probabilistic and statistical concepts about frequencies, associations and effects of health interventions. |
| **Communication of uncertainty in health,** | Strategies, encompassing different languages and formats, to communicate uncertainties about associations and effects of health interventions. |
| **Teaching/learning** | Education/learning strategies to inform and enhance the population's understanding of the meaning and relevance of key concepts in scientific evidence. |
| **Evidence synthesis frameworks using plain language** | Templates for evidence syntheses or other related documents in plain language, such as synthesis for policies and summaries of systematic reviews, made available as texts, infographics, printed or virtual (social media or websites). |
| **Guidelines for elaborating/evaluating communication products** | Guidelines, checklists and recommendations for developing and/or evaluating the quality of scientific evidence communication products for the population or managers. |
