# Supplementary material for: Strategies for communicating scientific evidence on healthcare to managers and the population: a scoping review
Source: Health Res Policy Syst. 2023 Jul 10;21:71. doi: 10.1186/s12961-023-01017-2 (PMC10334604; doi:10.1186/s12961-023-01017-2)
Supplement: Supplementary file 3 — Additional file 3. Studies/documents excluded and reasons for exclusions after reading the full text (second phase of the selection process). [file 12961_2023_1017_MOESM3_ESM.docx]

**Additional Material 3**. Studies/documents excluded and reasons for exclusions after reading the full text (second phase of the selection process).

|  | **Study/document (author/year)** | **Reason for exclusion** |
| --- | --- | --- |
| 1 | Adam 2014^17^ | Unsuitable content (does not address strategies for communicating scientific evidence in health to health managers and the population). It evaluates the nomenclature used in the literature for the term "policy brief". |
| 2 | AHRQ 2013^18^ | Unsuitable content (does not address strategies for communicating scientific evidence in health to health managers and the population). It reports the development of a catalogue of courses/educational strategies related to patient safety. |
| 3 | AlKhaldi 2021^19^ | Unsuitable content (does not address strategies for communicating scientific evidence in health to health managers and the population). It reports the difficulties and barriers identified in the health decision-making process. |
| 4 | Armstrong 2006^20^ | Unsuitable content (does not address strategies for communicating scientific evidence in health to health managers and the population). It reports theoretical and conceptual elements about the process of knowledge translation. |
| 5 | Armstrong 2013^21^ | Unsuitable content (does not address strategies for communicating scientific evidence in health to health managers and the population). It describes the design and implementation plan of a knowledge translation intervention for public health decision-making in local government. |
| 6 | Ashcraft 2020^22^ | Unsuitable content (does not address strategies for communicating scientific evidence in health to health managers and the population). It focuses exclusively on strategies for disseminating research on different health issues to policy makers. |
| 7 | Austvoll-Dahlgren 2016^23^ | Unsuitable content (does not address strategies for communicating scientific evidence in health to health managers and the population). It presents an overview of interventions on key concepts that people need to understand to evaluate treatments and identifies tools to assess understanding of these concepts. |
| 8 | Barac 2014^24^ | Unsuitable content (does not address strategies for communication of scientific evidence in health for health managers and the population). It reports the creation and maintenance of a website for the population with information on health topics, diseases and treatments in general. |
| 9 | Bastian 2005^25^ | Unsuitable content (does not address strategies for communication of scientific evidence in health for health managers and the population). It presents the results of a scoping review on tools (toolkits) used to inform health professionals and the population about health topics, diseases and treatments in general. |
| 10 | Bastian 2008^26^ | Unsuitable content (does not address strategies for communication of scientific evidence in health for health managers and the population). It presents the results of a scoping review on tools (toolkits) used to inform health professionals and the population about health topics, diseases and treatments in general. |
| 11 | Campbell 2019^27^ | Unsuitable population and content (does not address strategies for communication of scientific evidence in health for health managers and the population). It presents strategies to promote knowledge translation with a focus on health professionals. |
| 12 | Carman 2017^28^ | Unsuitable content (does not address strategies for communication of scientific evidence in health for health managers and the population). It presents conceptual aspects of the involvement of patients and families in research. |
| 13 | Carvalho 2015^29^ | Unsuitable content (does not address strategies for communication of scientific evidence in health for health managers and the population). It presents a narrative of the process and translation of knowledge adopted in Ghana. |
| 14 | Colquhoun 2016^30^ | Unsuitable population. It presents a strategy to promote knowledge translation with a focus on health professionals. |
| 15 | Crowley 2018^31^ | Unsuitable content (does not address strategies for communicating scientific evidence in health to health managers and the population). It presents a narrative and the costs of the knowledge translation process. |
| 16 | Erismann 2021^32^ | Unsuitable content (does not address strategies for communicating scientific evidence in health to health managers and the population). It presents strategies to bring researchers and managers closer in the process of knowledge translation. |
| 17 | Evans 2016^33^ | Unsuitable content (does not address strategies for communicating scientific evidence in health to health managers and the population). It is a book with concepts and examples on critical appraisal focused on health professionals and population with minimal prior knowledge of the theme. |
| 18 | FDA-NIH Biomarker Working Group 2016^34^ | Unsuitable content and population (does not address strategies for communicating scientific evidence in health to health managers and the population). It presents a glossary for health professionals. |
| 19 | Fadlallah 2017^35^ | Unsuitable content (does not address strategies for communicating scientific evidence in health to health managers and the population). It evaluates the use of narratives and storytelling about research on different health issues to guide decision making. |
| 20 | Fervers 2003^36^ | Unsuitable content (does not address strategies for communicating scientific evidence in health to health managers and the population). It presents a model booklet with guidelines on cancer care. |
| 21 | Gallagher 2011^37^ | Unsuitable content (does not address strategies for communicating scientific evidence in health to health managers and the population). It compares the use of positive and negative structured messages to encourage clinical practice. contains guidelines for practice and not on evidence communication. |
| 22 | Genova 2012^38^ | Unsuitable content (does not address strategies for communicating scientific evidence in health to health managers and the population). It presents a model for evaluating health communication material in general. |
| 23 | Glenton 2010^39^ | Unsuitable content (does not address strategies for communicating scientific evidence in health to health managers and the population). It presents an opinion poll on initial proposals for the Cochrane Plan Language Summary. |
| 24 | Gudi 2021^40^ | Unsuitable content (does not address strategies for communicating scientific evidence in health to health managers and the population). It presents a theoretical discussion on Plan Language Summaries. |
| 25 | Han 2013^41^ | Unsuitable content (does not address strategies for communicating scientific evidence in health to health managers and the population). It presents theoretical concepts on uncertainties in health with a focus on health professionals and the doctor-patient relationship. |
| 26 | Hesse 2010^42^ | Unsuitable content (does not address strategies for communicating scientific evidence in health to health managers and the population). It presents theoretical concepts about communication in oncology. |
| 27 | Hibbard 2016^43^ | Unsuitable content (does not address strategies for communicating scientific evidence in health to health managers and the population). It presents a discussion on strategies for involving the patient in health decision-making. |
| 28 | Hupert 2017^44^ | Unsuitable content and population (does not address strategies for communication of scientific evidence in health for health managers and the population). It presents a glossary for health professionals. |
| 29 | Jirjis 2005^45^ | Unsuitable content (does not address strategies for communication of scientific evidence in health for health managers and the population). It presents frameworks for communication of clinical practice. |
| 30 | Kelechi 2010^46^ | Unsuitable population (presents an evidence synthesis template for use by nurses). |
| 31 | LaRocca 2012^47^ | Unsuitable content (does not address strategies for communicating scientific evidence in health to health managers and the population). It compares different means to distribute clinical guidelines. |
| 32 | Lavis 2010^48^ | Unsuitable content (does not address strategies for communicating scientific evidence in health to health managers and the population). It describes the process of developing a series of policy briefs developed in Burkina Faso. |
| 33 | Moat 2014^49^ | Unsuitable content (does not address strategies for communicating scientific evidence in health to health managers and the population). It presents views and experiences on the use of policy briefs in low- and middle-income countries. |
| 34 | Nair-bedouelle 2021^50^ | Unsuitable content (does not address strategies for communicating scientific evidence in health to health managers and the population). It presents UNESCO discussion on how to improve scientific communication during the pandemic. |
| 35 | O'Keefe 2007^51^ | Unsuitable content (does not address strategies for communicating scientific evidence on health to health managers and the population). It compares the use of positive and negative structured messages to encourage disease prevention. Presents guidelines for practice and not communication of evidence. |
| 36 | O'Keefe 2009^52^ | Unsuitable content (does not address strategies for communicating scientific evidence on health to health managers and the population). It compares the use of positive and negative structured messages to encourage disease prevention. Presents guidelines for practice and not communication of evidence. |
| 37 | O'Keefe 2012^53^ | Unsuitable content (does not address strategies for communicating scientific evidence on health to health managers and the population). It compares the use of positive and negative structured messages to encourage disease prevention. Presents guidelines for practice and not communication of evidence. |
| 38 | Oxman 2021^54^ | Unsuitable population. It presents strategies for communicating scientific evidence in health to students and health professionals. |
| 39 | Pettman 2013^55^ | Unsuitable content (does not address strategies for communication of scientific evidence in health for health managers and the population). It presents the report of a training on types of evidence and critical appraisal to health managers. |
| 40 | Rapport 2017^56^ | Unsuitable content (does not address strategies for communication of scientific evidence in health for health managers and the population). It presents key concepts on implementation science and challenges faced by researchers in knowledge translation. |
| 41 | Sachs 2002^57^ | Unsuitable content (does not address strategies for communication of scientific evidence in health for health managers and the population). It addresses conceptual aspects of clinical communication between health professionals and patients. |
| 42 | Semakula 2019a^58^ | Unsuitable content (does not address strategies for communication of scientific evidence in health for health managers and the population). It describes the process of building the podcast used in the clinical trial reported in Semakula 2017 (already included in this review). |
| 43 | Sim 2002^59^ | Unsuitable content (does not address strategies for communication of scientific evidence in health for health managers and the population). It addresses informatics strategies to bring physicians closer to health evidence. |
| 44 | Soafer 2010^60^ | Unsuitable content (does not address strategies for communication of scientific evidence in health for health managers and the population). It is a guide to assist the consumer in classifying the quality of health services, such as choosing a doctor or hospital. |
| 45 | Solomon 2022^61^ | Unsuitable content (does not address strategies for communication of scientific evidence in health for health managers and the population). It is a guidance on the preparation of informed consent terms for clinical trials in an accessible format. |
| 46 | Teichman 2020^62^ | Unsuitable content (does not address strategies for communication of scientific evidence in health for health managers and the population). It assesses the number of posts on social media about clinical and practice recommendations related to the Covid-19 pandemic. |
| 47 | The SURE 2020^63^ | Unsuitable content (does not address strategies for communication of scientific evidence in health for health managers and the population). It presents ways to involve different stakeholders in the health policy process. |
| 48 | Thissen 2021^64^ | Unsuitable content (does not address strategies for communication of scientific evidence in health for health managers and the population). It reports an initiative to support managers in the process of economic impact assessment and social and health policies. |
| 49 | Yost 2015^65^ | Unsuitable content (does not address strategies for communication of scientific evidence in health for health managers and the population). It pesents strategies to facilitate the use of scientific evidence by nurses. |
| 50 | Yousefi 2020^66^ | Unsuitable content (does not address strategies for communication of scientific evidence in health for health managers and the population). It pesents strategies to implement equity and science centres. |
