# Supplementary material for: Strategies for communicating scientific evidence on healthcare to managers and the population: a scoping review
Source: Health Res Policy Syst. 2023 Jul 10;21:71. doi: 10.1186/s12961-023-01017-2 (PMC10334604; doi:10.1186/s12961-023-01017-2)
Supplement: Supplementary file 4 — Additional file 4. Main characteristics of included studies. [file 12961_2023_1017_MOESM4_ESM.docx]

**Additional Material 4.** Main characteristics of included studies.

| **Study (author, year)** | **Study design/publication type** | **Set of communiction trategies** | **Proposing institution** | **Financial source** |
| --- | --- | --- | --- | --- |
| **Antonopoulou 2021^68^** | Guideline/ on-line report | Guidelines for elaborating summaries of evidence for managers. | NIHR | NIHR |
| **Akl 2011a ^6^** | Systematic review / full-text article | Communication of health attributes as positive or negative.  Communicating the consequences of an action/behaviour, such as gains or losses. | Not applied. | State University of New York.  Italian National Cancer Institute.  Norwegian Research Council.  European Commission. |
| **Akl 2011b ^7^** | Systematic review / full-text article | Communication of health risks and benefits with different numerical or nominal forms. | Not applied. | None declared. |
| **Arienti 2018^69^** | Case study / full-text article | Blogshots for communicating content from systematic reviews. | Cochrane | None declared. |
| **Balshem 2011^70^** | Case study / full-text article | Inclusion of stakeholders in the group for preparing comparative effectiveness summaries. | AHRQ | AHRQ |
| **Baur 2014^71^** | Survey/ full-text article | Guidelines for designing and evaluating health communication products (CDC Clear Communication Index). | CDC | CDC |
| **Bireme 2020^72^** | Descriptive sudy / on-line bulletim | Description and presentation of evidence maps. | Bireme, Rede MTCI Américas, Consórcio Acadêmico Brasileiro de Saúde Integrativa (CABSIn), | Brazilian Ministry of Health and PAHO. |
| **Büchter** **2014^8^** | Systematic review / full-text article | Communication of health risks and benefits nominally versus numerically. | Not applied. | Department of Health Information, Institute for Quality and Efficiency in Health Care |
| **Büchter** **2020^73^** | RCT / full-text article | Communication of uncertainty about the effects of health interventions. | Institute for Quality and Efficiency in Health Care | Institute for Quality and Efficiency in Health Care |
| **Burkiewicz 2018^10^** | Narrative review / full-text article | Communication of risks and benefits of health interventions. | None declared. | None declared. |
| **Busert** **2018^74^** | Survey / full-text article | Summary template of systematic reviews in accessible language. | Cochrane Public Health Europe | Bavarian Health and Food Safety Authority. Lower Austria Health and Social Fund. Swiss School of Public Health |
| **Carroll 2006^75^** | Descriptive sudy / full-text article | Summary template of social and health policies in accessible language. | Trent Researchand Development Support Unit. Social Care Institute for Excellence. Social Care Access to Research Evidence. | Social Care Institute for Excellence. |
| **Castle 2017^76^** | Descriptive sudy / full-text article | Library on key concepts on health safety (CARL Library). | IHC. James Lind Initiative | Research Council of Norway  National Institute for Health Research. |
| **Chambers 2011^77^** | Scoping review/ full-text article | Evidence synthesis summary templates in accessible language. | Not applied. | None declared. |
| **Chapman 2020^2^** | Overview / full-text article | Communication of risks and benefits in health. | Not applied. | None declared. |
| **Cockcroft 2014^78^** | Case study / full-text article | Training for parliamentarians on scientific evidence in health. | National AIDS Coordinating Agency | Global Health Research Initiative |
| **Cusack 2018^9^** | Sytematic review / full-text article | Communication/learning of key concepts about health evidence. | Not applied. | None declared. |
| **Davidson 2017^79^** | Narrative review / full-text article | Narratives and storytelling for communicating evidence for decision-making in public policies. | None declared. | None declared. |
| **Ebell 2004a^80^ / Ebell 2004b^81^** | Descriptive sudy / full-text article | Assessment and communication tool for quality, quantity and consistency of evidence in health (SORT). | Consortium of journals specializing in Family Medicine in the United States. | None declared. |
| **Edwards 2001^82^** | Sytematic review / full-text article | Communication of risks and benefits of health interventions. | Not applied. | UK National Health Service Research and Development programme |
| **Epstein 2004^83^** | Sytematic review / full-text article | Communication of risks and benefits in health. | Not applied. | None declared. |
| **ERA-ENVHEALTH 2019^84^** | Diretriz / on-line report | Guidelines for the development of products for the communication of scientific results used in the formulation of policies. | ERA-ENVHEALTH | European Community's Seventh  Framework Programme |
| **Fortin 2001^85^** | Survey / full-text article | Communication of risks and benefits in health. | None declared. | None declared. |
| **Freeman 2021a^86^/ Freeman 2021b^87^** | Descriptive study / conference abstract | Communication of evidence on Covid-19. | Winton Centre for Risk & Evidence Communication. University of Cambridge. Harding Center for Risk Literacy, University of Potsdam. | None declared. |
| **Ghosh 2005^88^** | Narrative review / full-text article | Communication of risks and benefits in health. | None declared. | None declared. |
| **Ghosh 2008^89^** | ECR/ full-text article | Communication of health risks and benefits with graphs or with graphs and diagram of frequency. | None declared. | Medicine Innovation Development and Advancement System. |
| **Gigerenzer 2007^90^** | Descriptive study / full-text article | Communication of risks and benefits in health. | None declared. | None declared. |
| **Glenton 2020^91^** | Guideline/ on-line report | Guidelines for the elaboration of products to communicate the results of systematic reviews. | Cochrane | Cochrane |
| **Grimshaw 2012^11^** | Narrative review / full-text article | Communication of risks and benefits in health. | Not applied. | None declared. |
| **Hartling 2018^92^** | Survey / on-line report | Evidence synthesis summary templates in accessible language. | AHRQ | AHRQ |
| **IHC 2018^93^** | Descriptive sudy / on-line report | Communication/learning of key concepts on health evidence. | IHC | Research Council of Norway. National Institute for Health Research. Rockefeller Foundation. Naji Foundation. Norwegian Agency for Development Cooperation. European Union. |
| **Ikirezi 2016^94^** | Case study / on-line report | Comunicação/ aprendizagem de conceitos-chave sobre evidências em saúde. | IHC | Norwegian Research Council . |
| **Kerwer 2021^95^** | Survey/ full-text article | Abstract template in accessible language. | Journal of Social and Political Psychology | Open Access Fund of the Leibniz Association. |
| **Knapp 2004^96^** | ECR / full-text article | Communication of health risks and benefits using nominal or numerical forms. | None declared. | None declared. |
| **Krause 2011^97^** | Narrative review / conference abstract | Interactive communication/learning of key concepts on health evidence. | Not applied. | Colorado School of Public Health. Dartmouth Institute for Health Policy and Clinical Practice. Norwegian Knowledge Center for the Health Services |
| **Kristiansen** **2012^98^** | Survey / full-text article | Communication of risks and benefits in health.  using NNT. | None declared. | Danish Heart Foundation |
| **Lavis 2013^99^** | Descriptive sudy / on-line report | Guidelines for the development of communication products on health policies and systems in accessible language. | BRIDGE Study Team | WHO Regional Office for Europe and European Observatory on Health Systems and Policies |
| **Lipkus 2007^100^** | Narrative review / full-text article | Communication of risks and benefits in health. | Not applied. | AHRQ. Foundation for Informed Medical Decision Making. |
| **Logullo 2019^101^** | Translation and transcultural adaptation study/ full-text article | Tool to assess the quality of health texts. | University of Oxford. British Library | CAPES |
| **Lopez 2008^102^** | Sytematic review / full-text article | Communication of risks and benefits in health. | Not applied. | None declared. |
| **Marquez 2018^103^** | Survey / full-text article | Systematic review templates in accessible language. | CIHR | CIHR |
| **McCormack 2013^1^** | Sytematic review  / on-line report | Communication of risks and benefits in health. | Not applied. | AHRQ |
| **Medendorp 2021^12^** | Scoping review / full-text article | Guidelines for communicating uncertainties in accessible language. | Not applied. | Dutch Cancer Society. Dutch Research Council. |
| **Moberg 2018^104^** | Descriptive sudy / full-text article | Glossary (GET-IT) of health research terms. | GRADE. IHC. Testing Treatments interactive. | Research Council of Norwa. European Commission under the Seventh Framework Programme. |
| **Moretti 2018^105^** | Descriptive sudy / full-text article | E-book with summaries of systematic reviews in accessible language. | Cochrane | Cochrane |
| **Mosconi 2016^106^** | Descriptive sudy / full-text article | Communication/ learning about Clinical Trials (ECRAN Project). | Projeto ECRAN | European Union’s Seventh Framework Programme |
| **Mugisha 2016^107^** | Case study / full-text thesis | Communication/learning of key concepts on health evidence. | IHC | Research Council of Norway |
| **Murthy 2012^108^** | Sytematic review / full-text article | Health evidence communication newsletters in accessible language. | Not applied. | Cochrane |
| **Nordheim 2016^109^** | Sytematic review / full-text article | Communication/learning of key health evidence concepts. | Not applied. | None declared. |
| **Nsangi 2017^110^** | Clinical trial / full-text article | Communication/learning of key concepts about health evidence. | IHC | Research Council of Norway |
| **Ongolo-Zogo 2014^111^** | Survey / full-text article | Communication/learning of key concepts about health evidence. | EVIPNet de Camarões e a REACH-PI de Uganda. | None declared. |
| **Oxman 2020a^112^** | Descriptive sudy / full-text article | Guidelines for communicating evidence on the effects of health interventions. | Centre for Informed Health Choices, Norwegian Institute of Public Health, Oslo, Noruega. | Centre for Informed Health Choices, Norwegian Institute of Public Health. |
| **Oxman 2020b^113^** | Sytematic review / full-text article | Communication/learning of key concepts on health evidence for the population. | Not applied. | Research Council of Norway |
| **Paling 2003^114^** | Descriptive sudy / full-text article | Communication of health risks (CARE approach). | None declared. | None declared. |
| **Parmelli 2022^115^** | Descriptive sudy / full-text article | Communication of certainty of evidence to managers (DECIDE Project). | GRADE | European Commission under the Seventh Framework Programme |
| **Petkovic 2016^116^** | Sytematic review / full-text article | Evidence synthesis summary templates in accessible language. | None declared. | Nenhuma declarada. |
| **Prictor 2013^117^** | Case study / full-text article | Patient involvement in the Cochrane systematic review process. | Cochrane | Cochrane |
| **Puljak 2015^118^** | Case study / full-text article | Use of Facebook to communicate health evidence. | Cochrane Croácia | Cochrane |
| **Ringle 2020^119^** | Descriptive sudy / on-line dissertaion | Educational podcasts for communication/learning of key concepts about health evidence. | None declared. | None declared. |
| **Robinson 2005^120^** | Descriptive sudy / resumo | Communication/learning about clinical trials. | None declared. | None declared. |
| **Rosenbaum 2011^121^** | Case study / full-text article | Summary template of systematic reviews in accessible language. | SUPPORT | European Commission’s 6th Framework Programme |
| **Ryan 2018^122^** | Case study / full-text article | Use of Facebook and Twitter to communicate health evidence. | None declared. | None declared. |
| **Santesso 2006^123^** | Case study / full-text article | Communicating the contents of systematic reviews. | Cochrane | CADTH. Canadian Institutes of Health Research. |
| **Santesso 2015^124^** | RCT/ full-text article | Evidence summary template in accessible language. | Cochrane | Canadian Institutes of Health Research. |
| **Semakula 2017^125^/ Semakula 2020^126^** | RCT/ full-text article | Educational podcasts for communication/learning of key concepts about health evidence. | IHC | Research Council of Norway, |
| **Semakula 2019b^127^** | Descriptive sudy / full-text article | Educational podcasts for communication/learning of key concepts about health evidence. | IHC | Research Council of Norway, |
| **Sheridan 2003^128^** | RCT / full-text article | Strategies for communicating risks and benefits in health. | None declared.  . | None declared. |
| **The SHARE Approach 2020^129^** | Descriptive sudy / full-text report | Guide for communicating numerical health evidence results. | AHRQ | AHRQ |
| **Trevena 2006^130^** | Sytematic review / full-text article | Communication of risks and benefits in health. | Not applied. | None declared. |
| **WAHO**  **2021^131^** | Guideline / on-line report | Guidelines for the elaboration of evidence syntheses in health policies for managers. | WAHO | WAHO |
| **Welch 2013^132^** | Descriptive sudy / full-text article | Guidelines for communicating the results of systematic reviews in accessible language. | Campbell and Cochrane Equity Methods Group | Canadian Institutes of Health Research |
| **Wickremasinghe 2015^133^** | Narrative review / full-text article | Evidence synthesis summary templates in accessible language. | Not applied. | PMNCH Knowledge Summary series (2013/346244) |
| **Woloshin 2003^134^** | Descriptive sudy / full-text article | Guidelines for communicating evidence (including probabilities, risks and benefits). | None declared. | None declared. |
| **Woloshin 2008^135^** | Guideline/ on-line report | Guidelines for communicating evidence (including probabilities, risks and benefits). | University of California | University of California |
| **Woolf 2015^136^** | Sytematic review / full-text article | Guidelines for communicating evidence in health. | Not applied. | Robert Wood Johnson Foundation. NIH. |
| **Zikmund-Fisher 2012^137^** | RCT / full-text article | Communication of health risks and benefits with animated graphics. | Not applied. | Foundation for Informed Medical Decision Making. |
| **Zikmund-Fisher 2013^138^** | Descriptive sudy / full-text article | Guidance for communicating analysis (including probabilities, risks and benefits). | Not applied. | American Cancer Society |

*AHRQ: Agency for Healthcare Research and Quality; Bireme: Biblioteca Regional de Medicina; CADTH: Canadian Agency for Drugs and Tehnologies in Health; CAPES: Coordenação de Aperfeiçoamento de Pessoal de Nível Superior; CARL: Canadian Association of Research Libraries; CDC: Centers for Disease Control and Prevention; CIHR: Canadian Institutes of Health Research; ERA-ENVHEALTH: Research Area Network for Environment and Health; DECIDE: Developing and Evaluating Communication Strategies to Support Informed Decisions and Practice Based on Evidence; ECRAN: European Communication on Research Awareness Need; ERIC: Education Resources Information Center; EVIPNet: Evidence informed policy network; GET-IT: Glossary of Evaluation Terms for Informed Treatment choices; GRADE: Grading of Recommendations Assessment, Development and Evaluation; IHC: Informed Health Choices; MEDLINE: Medical Literature Analysis and Retrievel System Online; NIH: National Institute of Health; NIHR: National Institute for Health and Care Research; NNT: number needed to treat; PAHO: Pan-American Health Organization; PMNCH: Partnership for Maternal, Newborn and Child Healt; RCT: randomized clinical trial; REACH-PI: Regional East African community health policy initiative; ARR: absolute risk reduction; RRR: relative risk reduction; SORT: Strength of Recommendation Taxonomy; SUPPORT: Supporting Policy-relevant Reviews and Trials; WAHO West African Health Organization; WHO: World Health Organization.*
