# Supplementary material for: Strategies for communicating scientific evidence on healthcare to managers and the population: a scoping review
Source: Health Res Policy Syst. 2023 Jul 10;21:71. doi: 10.1186/s12961-023-01017-2 (PMC10334604; doi:10.1186/s12961-023-01017-2)
Supplement: Supplementary file 5 — Additional file 5. Main characteristics of the identified strategies or sets of strategies for communicating scientific evidence. [file 12961_2023_1017_MOESM5_ESM.docx]

**Additional material 5**. Main characteristics of the identified strategies for communicating scientific evidence.

| **Strategy** | **Description** | **Category** | **Target audience** | **Focus** | **Delivery approach** | **Status** | **Barriers** | **Facilitators** |
| --- | --- | --- | --- | --- | --- | --- | --- | --- |
| **Guidelines for the development of communication products [Antonoupoulos 2021]^68^** | Checklist with minimum items to guide the elaboration of health policy summaries. | Guidelines for the development/ assessment of communication products | Managers | Language Content Format | Textual Visual  Verbal | Implemented and not assessed | NR | NR |
| **Communicating health characteristics as positive or negative**  **[Akl 2011a]^6^** | Communicating risks or chances of health characteristics as positive or negative.  Exemple: “the chance of survival with canceris 2/3” versus “the chance of mortality with cancer is 1/3”. | Risk/benefit communication | Population | Language | Textual  Verbal | Implemented and assessed | NR | NR |
| **Communication of health outcomes**  **[Akl 2011a]^6^** | Communication of the consequences of na action / behavior in health as benefit (positive) or as reduction (negative).  Exemple: “if you undergo a screening test for cancer, your survival will be prolonged” *versus* “if you don’t undergo screening test for cancer, your survival will be shortened”. | Risk/benefit communication | Population | Language | Textual  Verbal | Implemented and assessed | NR | NR |
| **Communication of risks / benefits in health**  **[Akl 2011b]^7^** | Communication of risks in health using different methods: (i) frequency (5 in 100) *versus* percentage (5%); (ii) RRR *versus* ARR versus NNT. | Risk/benefit communication | Population | Language | Textual  Verbal | Implemented and assessed | NR | NR |
| **Blogshots for communicating content from systematic reviews [Arienti 2018]^69^** | Blogshots (visual presentation with an infographic on a single slide, on social media with the possibility of sharing and comments) to facilitate access and understanding of scientific evidence from systematic reviews.  Content: (i) a representative image of the intervention or condition; (ii) the logo of Cochrane or the group involved; (iii) the title of the review; (iv) key findings and/or implications for practice; (v) standardized reporting of the certainty of evidence; (vi) informative sentence with the number of studies and participants included in the review, the intervention and the evaluated comparator; (vii) the Cochrane editorial group that produced the review; (viii) link to the full review. | Evidence synthesis templates or other documents in accessible language | Population | Language  Content  Format | Textual  Visual (gráfica) | Implemented and assessed | NR | NR |
| **Inclusion of stakeholders in the group for preparing comparative effectiveness summaries**  **[Balshem 2011]^70^** | Inclusion of stakeholders in the group to prepare comparative effectiveness summaries produced by the AHRQ to improve the communication of content when the evidence is weak, uncertain or conflicting. | Evidence synthesis templates or other documents in accessible language | Managers | Language  Content  Format | Textual | Implemented and assessed | Stakeholder time availability.  Stakeholders' in-depth knowledge of the purpose and methods of the summary.    Additional resources for stakeholder inclusion in co-authorship. | Interaction between the group and developers. |
| **Guidelines for designing and evaluating health communication products (CDC Clear Communication Index)**  **[Baur 2014]^71^** | The CDC Clear Communication Index is a checklist to guide the design and evaluation of health communication products for different audiences. The checklist consists of 4 introductory questions and 20 items selected from the scientific literature in communication and related disciplines, representing the most important aspects that improve and help in the understanding of the information. (http://www.cdc.gov/healthcommunication/ClearCommunicationIndex/). | Guidelines for the development/ assessment of communication products | População  Gestores | Language  Content  Format | Textual (on-line) | Implemented and assessed | NR | NR |
| **Evidence maps [Bireme 2020]^72^** | Evidence maps (n=14) on integrative practices | Evidence synthesis templates or other documents in accessible language | Managers | Language  Content  Format | Textual  (*on-line*) | Implemented and not assessed | High speed of publication of new studies. | Ease of access (online) |
| **Communication of health risks nominally or numerically**  **[Büchter 2014]^8^** | Communication of risk of adverse events associated with treatments nominally ('common', 'uncommon' and 'rare') versus numerically (percentage, frequency). | Risk/benefit communication | Population | Language | Textual | Implemented and assessed | NR | NR |
| **Communication of uncertainty about the effects of health interventions**  **[Büchter** **2020]^73^** | Eight versions of a summary of the effects of a fictional tinnitus medication were compared. The versions differed in the way of presenting the degree, the reasons and the magnitude of the uncertainty. | Comunicação de incerteza | Population | Language  Content | Textual | Implemented and assessed | NR | NR |
| **Communication of risks and benefits of health interventions for the population [Burkiewicz 2018]^10^** | Strategies to communicate risks and benefits of health interventions to the population within the following topics: (i) how to present evidence to patients, (ii) communication of general terms, (iii) communication of risks and use of numbers, (iv) tips for communicating evidence. | Risk/benefit communication | Population | Language  Content | Textual  Verbal | Implemented and not assessed | NR | NR |
| **Systematic review summary templates**  **[Busert** **2018]^74^** | Systematic reviews summary template adapted for managers with the following content and structure: (i) title; (ii) table with primary information: a brief introduction, results, number of studies and participants included; (iii) framework with 'public health relevance'; (iv) table with review inclusion criteria and characteristics of included studies; (v) table explaining the GRADE; (vi) Results of the findings summary tables; (vii) tables with information on subgroup effects, study funding and conflict of interest and additional information; (viii) glossary of technical terms. | Evidence synthesis templates or other documents in accessible language | Managers | Language  Content  Format | Textual | Implemented and not assessed | NR | NR |
| **Social and health policy synthesis template [Carroll 2006]^75^** | Synthesis model for managers following the process (i) definition of topic and scope through discussion and literature search; (ii) elaboration of the first version based on the literature identified by structured searches, and of quality, producing an accurate summary of the findings. (iii) peer review of the text using a standardized form that assesses information accuracy, adequacy to the criteria established for the topic and scope, usefulness for the target population, inclusion of relevant ethical and legal issues, accessibility if there was a loss of some important document; (iv) consultation with information consumers. (v) final review of the abstract and publication with the main findings of the abstract. | Evidence synthesis templates or other documents in accessible language | Managers | Language  Content  Format | Textual | Implemented and not assessed | Identification of themes that are common to the social and health areas | NR |
| **Library of key concepts on health evidence (CARL Library)**  **[Castle 2017]^76^** | Online library with key concepts on health evidence to support critical thinking. CARL Library Contents: (i) Informed Health Choices (www.informedhealthchoices.org); (ii) Testing treatments (www.testingtreatments.org); (iii) The James Lind Library (www.jameslindlibrary.org); (iv) Systematic reviews of educational interventions; (v) Educational Endowment Foundation (www.educationendowmentfoundation.org.uk); (vi) Times Education Service (www.tes.com/teaching-resources); (vii) Searches in electronic databases such as ERIC and MEDLINE. | Teaching/  learning | Population | Language  Content | Textual (on-line) | Implemented and not assessed | Difficulty in measuring the impact of access to the CARL Library. Restricted to the English language. | NR |
| **Evidence synthesis summary template**  **[Chambers 2011]^77^** | Summary templates for systematic reviews, overviews and syntheses of health policies, produced by different organizations. | Evidence synthesis templates or other documents in accessible language | Managers | Language  Content  Format | Textual | Implemented and not assessed | Growing volume of information and limited resources. | NR |
| **Communication of risks and benefits in health**  **[Chapman 2020]^2^** | Strategies for knowledge translation and among them some on the communication of evidence in health, including communication of risks and benefits in health. | Risk/benefit communication | Population | Language  Content  Format | Textual  Visual  Verbal | Implemented and assessed | Lack of health literacy or literacy with electronic media. | Social media can expand access to health information for ethnic minorities and lower socioeconomic groups. |
| **Training for parliamentarians on scientific health evidence**  **[Cockcroft 2014]^78^** | Theoretical-practical training for Botswana parliamentarians on health evidence. | Teaching/  learning | Managers | Language  Content  Format | Textual Visual  Verbal | Implemented and assessed | New content and conflicts of interest. | Use of local examples and small number of managers |
| **Communication/learning of key concepts about health evidence**  **[Cusack 2018] ^9^** | Communication/learning strategies are carried out inside and outside the school environment, single or multiple, using different approaches such as discussion groups, printed material, online classes, and short or long-term courses. The outcomes evaluated included: knowledge, skill, behaviour, confidence, perception of knowledge and/or skill, attitude and satisfaction. | Teaching/  learning | Population | Language  Content  Format | Textual Visual  Verbal | Implemented and assessed | NR | NR |
| **Narratives and storytelling for communicating evidence [Davidson 2017] ^79^** | Different forms of narratives and storytelling to communicate evidence for decision-making in public policies. | Evidence synthesis templates or other documents in accessible language | Managers | Language  Content | Verbal | Implemented and not assessed | NR | NR |
| **Assessment and communication tool for the quality of evidence in health (SORT) [Ebell 2004a^80^/ Ebell 2004b^81^]** | Tool to assess the quality, quantity and consistency of results from primary studies or evidence syntheses focusing on patient-oriented outcomes (SORT). The criteria evaluated are (i) study quality (according to the design): graded at level 1, 2 or 3; (ii) consistency between studies: consistency or inconsistency and (iii) strength of recommendation (quality and consistency): graded in A, B or C. | Guidelines for the development/ assessment of communication products | Population | Content | Textual | Implemented and not assessed | NR | NR |
| **Communication of risks and benefits in health [Edward 2001]^82^** | Different forms of communication of health risks and benefits: nominal or numerical (proportion or absolute frequency), such as graphics or text. | Risk/benefit communication | Population | Language  Content  Format | Textual  Visual | Implemented and assessed | NR | NR |
| **Communication of evidence in health [Epstein 2004]^83^** | Different forms of communication of risks and benefits in health: not quantitative, numerical or graphic. | Risk/benefit communication | Population | Language  Content  Format | Textual Visual  Verbal | Implemented and assessed | NR | NR |
| **Guidelines for the development of evidence communication products**  **[ERA-ENVHEALTH 2019]^84^** | Checklist with minimum items to guide the development of evidence communication products. | Guidelines for the development/ assessment of communication products | Managers | Language  Content  Format | Textual | Implemented and not assessed | NR | NR |
| **Communication of risks and benefits in health [Fortin 2001]^85^** | Different forms of risk communication were evaluated by a sample of 15 premenopausal women. | Risk/benefit communication | Population | Language Format | Textual Visual | Implemented and assessed | Evaluated in a specific context of hormone replacement | NR |
| **Communication of uncertainties in evidence about Covid-19 [Freeman 2021a^86^/ Freeman 2021b ^87^]** | Strategies for reporting risks, uncertainty about the effects of interventions for Covid-19, and quality of evidence. The authors propose some guidelines when communicating evidence: (i) listen to the target audience (what information they need, what decision they are making), (ii) provide an appropriate and balanced context (do not ignore inconvenient evidence), (iii) ) be balanced in the way you present evidence as well as in the information itself (be aware of the effects of different formats, frameworks, etc.), (iv) be honest about uncertainties and how they are being resolved, (v) communicate the quality of evidence, (vi) avoid misunderstandings or misinformation. | Uncertainty communication | PopulationManagers | Language  Content  Format | Textual Visual  Verbal | Implemented and not assessed | NR | NR |
| **Communication of risks and benefits in health [Ghosh 2005]^88^** | Different forms of communication of health risks and benefits: nominal or numerical (proportion or absolute frequency), such as graphics or text. | Risk/benefit communication | Population | Language  Content  Format | Textual Visual | Implemented and assessed | NR | NR |
| **Communication of risks and benefits in health [Ghosh 2008]^89^** | Communication of health risks and benefits for women at high risk of breast cancer: with graphs or frequency graphs and diagrams. | Risk/benefit communication | Population | Language  Content  Format | Visual | Implemented and assessed | NR | NR |
| **Risk communication and other statistical health information [Gigerenzer 2007]^90^** | Risk communication and other health statistical information: (i) use of frequency rather than single event probabilities; (ii) use of absolute risks rather than relative risks; (iii) use of mortality instead of survival, (iv) use of natural frequencies instead of conditional probabilities. | Risk/benefit communication | Population | Language  Content  Format | Textual  Visual | Proposed | NR | NR |
| **Guidelines for the development of products for communicating the results of systematic reviews [Glenton 2020] ^91^** | Checklist with minimum items to guide the development of communication products based on Cochrane systematic reviews, templates for preparing summaries in plain language (plain language summaries), blogshots and summaries of health policies. | Guidelines for the development/ assessment of communication products | PopulationManagers | Language  Content  Format | Textual Visual  Verbal | Implemented and not assessed | NR | NR |
| **Communication of risks and benefits in health [Grimshaw 2012]^11^** | Communication strategies aimed at the population or managers are divided into categories according to objectives: facilitating communication and/or decision-making, supporting behaviour change, and informing and educating. | Risk/benefit communication | Population | Language  Content  Format | Textual Visual  Verbal | Implemented and assessed | NR | NR |
| **Evidence synthesis summary template [Hartling 2018]^92^** | Template and guidelines for preparing a summary of evidence synthesis for managers based on a survey conducted by AHRQ. | Evidence synthesis templates or other documents in accessible language | Managers | Language  Content  Format | Textual | Implemented and assessed | Ability to prepare summaries and availability of hours. | NR |
| **Communication/learning of key concepts about health evidence [IHC 2018]^93^** | Communication/learning Strategies for key concepts on health evidence to support critical thinking in health: (i) list of key concepts to support understanding of the effects of health interventions; (ii) tools to assess an individual's ability to apply these key concepts; (iii) learning resources to enable primary school children and their parents to understand and apply some of the key concepts about health evidence; (iv) a database of learning resources designed to help people understand and apply one or more key concepts; (v) glossary of health research terms in accessible language. | Teaching/  learning | Population | Language  Content  Format | Textual (on-line) | Implemented and not assessed | NR | NR |
| **Communication/learning of key concepts about health evidence [Ikirezi 2016]^94^** | Learning resources developed by the IHC initiative to support the understanding and application of key concepts on critical assessment of the evidence in health were implemented in a kindergarten in Rwanda to assess their feasibility. | Teaching/  learning | Population | Language  Content  Format | Textual Visual  Verbal | Implemented and assessed | Availability of time, training and teacher literacy. | Presence of facilitators stimulated the use of resources by children and their parents. |
| **Abstract template in accessible language [Kerwer 2021]^95^** | Abstract template with evidence and results of studies/articles prepared in accessible language. | Evidence synthesis templates or other documents in accessible language | Population | Language  Content  Format | Textual | Implemented and assessed | Only abstracts in the area of psychology were valid | NR |
| **Health risk communication using nominal or numerical forms**  **[Knapp 2004]^96^** | Communication of health risks using nominal (example: common, rare) or numerical forms (example: 2.5%, 0.15%). | Risk/benefit communication | Population | Language  Format | Textual | Implemented and assessed | NR | NR |
| **Interactive communication/learning tools for key concepts on evidence in health**  **[Krause 2011]^97^** | Interactive, free, online or downloadable tools to support critical thinking in health teach the population to interpret information about the effects of health care. | Teaching/  learning | Population | Language  Content  Format | Textual Visual  Verbal | Implemented and not assessed | Internet access to use or download tools.; need for continuous updating. | Free tools. |
| **Communication of health risks using the NNT [Kristiansen**  **2012]^98^** | The risk of infarction with a dummy drug was communicated through the NNT, and 675 individuals responded that the drug reduced the risk of infarction. | Risk/benefit communication | Population | Language  Content | Textual | Implemented and assessed | NR | NR |
| **Guidelines for the development of communication products in health policies and systems [Lavis 2013]^99^** | Format, content, and language guidelines for designing health communication products, including primary study summaries, systematic review summaries, policy summaries, summary compendia, and dialogue summaries. | Guidelines for the development/ assessment of communication products | Managers | Language  Content  Format | Textual | Implemented and not assessed | Need for translation into different languages. | NR |
| **Communication of risks and benefits in health [Lipkus 2007]^100^** | Communication of health risks and benefits using nominal, numerical or graphic forms. | Risk/benefit communication | Population | Language  Content  Format | Textual  Visual  Verbal | Implemented and assessed | NR | NR |
| **DISCERN tool to assess the quality of health texts [Logullo 2019]^101^** | Translation and adaptation of the DISCERN tool into Portuguese (http://www.discern.org.uk/discern_instrument.php). | Guidelines for the development/ assessment of communication products | Population | Content | Textual | Implemented and assessed | Available in multiple languages. | NR |
| **Communication of contraceptive efficacy [Lopez 2008]^102^** | Different strategies to communicate the effectiveness of contraceptives to the population were compared. | Risk/benefit communication | Population | Language  Content  Format | Textual | Implemented and assessed | Limited to the contraception scenario. | NR |
| **Systematic review summary templates [Marquez 2018]^103^** | Two models of systematic reviews summaries were compared to the traditional model. | Evidence synthesis templates or other documents in accessible language | Managers | Language  Content  Format | Textual | Implemented and assessed | NR | NR |
| **Communication of risks and benefits in health [McCormack 2013]^1^** | Different risk/benefit communication strategies, focusing on language, content and/or format. | Risk/benefit communication | Population | Language  Content  Format | Textual | Implemented and assessed | NR | NR |
| **Uncertainty communication strategies [Medendorp 2021]^12^** | Model for individual verbal communication between healthcare professionals and patients about healthcare uncertainties with the following content: (i) openly acknowledging inherent uncertainty and explaining the degree and nature of available evidence, (ii) allowing flexibility to the extent that uncertainty is communicated, depending on the individual and circumstances, (iii) describe all potential scenarios and discuss their implications for the patient's life; (iv) explain the uncertainty in an understandable, concrete and structured way, (v) use non-verbal communication that conveys confidence, (vi) verify the patient's understanding of the uncertainty. | Uncertainty communication | Population | Language  Content | Verbal | Implemented and not assessed | NR | NR |
| **Glossary (GET-IT) of health search terms**  **[Moberg 2018]^104^** | Glossary GET-IT, with terms in accessible language, developed by the GRADE working group as part of the DECIDE project. Available at: www.getitglossary.org). | Teaching/  learning | PopulationManagers | Language  Content | Textual  (*on-line)* | Implemented and not assessed | Available in only a few languages. | Online access and continuous update |
| **E-book with systematic reviews summaries in accessible language [Moretti 2018]^105^** | E-book with abstracts of Cochrane systematic reviews in the area of rehabilitation, available in accessible language for different audiences (population, health professionals, managers). (https://rehabilitation.cochrane.org/) | Evidence synthesis templates or other documents in accessible language | Population | Language  Content  Format | Textual  (on-line) | Implemented and not assessed | Available in English only. | Online access |
| **Communication/ learning strategies about clinical trials [Mosconi 2016]^106^** | The set of online communication/learning strategies called the ECRAN project, comprises: (i) a website (http://ecranproject.eu) in six languages, including a media section to help journalists disseminate information about the ECRAN project; (ii) animation about clinical trials, dubbed into the 23 official languages of the European Community, and an interactive tutorial; (iii) resource inventory, available in 23 languages, with search by topic, author, and media type; (iv) educational games for young people, developed in six languages; (v) interactive secondary website on treatment evaluation, available in 12 languages; and (vi) interactive slideshow to assess users' knowledge of clinical trials. | Teaching/  learning | Population | Language  Content  Format | Textual  Visual  Verbal  (on-line) | Implemented and not assessed | NR | NR |
| **Communication/ learning of key concepts about health evidence [Mugisha 2016]^107^** | Communication/learning resources developed by the IHC initiative to support the understanding and application of key concepts on critical assessment of the evidence in health were applied in a kindergarten in Rwanda. | Teaching/  learning | Population | Language  Content  Format | Textual Visual  Verbal | Implemented and assessed | Availability of time, training and literacy of teachers. | The presence of facilitators stimulated the use of resources by children and their parents. |
| **Newsletters for communication of health evidence**  **[Murthy 2012]^108^** | Printed newsletters for communicating health evidence to the population. | Evidence synthesis templates or other documents in accessible language | Population | Language  Content  Format | Textual | Implemented and assessed | NR | NR |
| **Communication/ learning of key concepts about health evidence [Nordheim 2016]^109^** | Communication/learning resources to enable students in primary, secondary, or other equivalent educational institutions to understand and apply key concepts in critically evaluating evidence in health. | Teaching/  learning | Population | Format | Textual  Verbal | Implemented and assessed | Low student adherence; inadequate prior knowledge about health evidence; teachers' literacy. | NR |
| **Communication/learning of key concepts about health evidence [Nsangi 2017]^110^** | Communication/learning resources developed by the IHC initiative to support the understanding and application of key concepts in critically assessing evidence in health were applied at a preschool in Uganda. | Teaching/  learning | Population | Language  Content  Format | Textual Visual  Verbal | Implemented and assessed | Availability of time, training and teacher literacy. | Presence of facilitators stimulated the use of resources by children and their parents. |
| **Communication of evidence in health [Ongolo-Zogo 2014]^111^** | Strategies for knowledge translation and communication of evidence in health for the population and health policy managers in Cameroon and Uganda. | Teaching/  learning | PopulationManagers | Language  Content  Format | Textual Visual  Verbal | Implemented and assessed | NR | NR |
| **Evidence communication checklist on the effects of health interventions [Oxman 2020a]^112^** | Evidence communication checklist on the effects of health interventions with ten recommendations: three to facilitate and speed up the identification of topic relevance and key messages; five to promote understanding of the size of effects and certainty in estimates; two to help the reader contextualize information about the effects of the intervention and understand why the information is reliable. | Evidence synthesis templates or other documents in accessible language | Population | Content | Textual | Implemented and not assessed | NR | NR |
| **Communication/ learning of key concepts about health evidence [Oxman 2020b]^113^** | Twenty-two learning strategies to support understanding and applying key concepts about critical assessment of evidence in health to stimulate critical thinking. | Teaching/  learning | Population | Language  Content  Format | Textual Visual  Verbal | Implemented and not assessed | NR | NR |
| **Communication of risks and benefits in health (CARE approach) [Paling 2003]^114^** | The CARE approach to communicating risks and benefits in health to the population consists of C: citing the risk in descriptive terms ('high risk' or 'low risk') to give context to the outcome; A: adding the probabilities (presenting the evidence in numbers, as in the example: 15 out of 100 people may experience this adverse event); R: reinforce with visual examples (graphs, risk scales and perspective scale, faces of joy and sadness, colours of traffic lights); E: expressing support (reinforcing the components of care related to the patient and making oneself available). | Risk/benefit communication | Population | Language  Content | Visual  Verbal | Implemented and not assessed | NR | NR |
| **Evidence certainty communication strategy (DECIDE project)**  **[Parmelli 2022]^115^** | The GRADE working group led the DECIDE project (2011 to 2015) intending to improve the dissemination of evidence-based recommendations (https://www.decide-collaboration.eu/). Among the DECIDE tools related to communication for the population and managers, this article presents the GRADE Evidence to Decision (https://www.decide-collaboration.eu/evidence-decision-etd-framework). | Risk/benefit communication | Managers | Language  Content  Format | Textual | Implemented and not assessed | Concurrent update to the GRADE summary of findings table. | NR |
| **Systematic review summary templates [Petkovic 2016]^116^** | Use of summary models of systematic reviews in healthcare decision making. | Evidence synthesis templates or other documents in accessible language | Managers | Language  Content  Format | Textual | Implemented and assessed | NR | NR |
| **Inclusion of patients in the process of preparing systematic reviews**  **[Prictor 2013]^117^** | Patient involvement in preparing Cochrane systematic reviews to improve communication, understanding and patient engagement in healthcare decision-making. | Teaching/  learning | Population | Language  Content  Format | NA | Implemented and not assessed | Difficulty in measuring the use of summaries in decision making | NR |
| **Use of Facebook to communicate health evidence**  **[Puljak 2015]^118^** | Creation of a Facebook page by the Cochrane Croatia team to communicate abstracts of Cochrane systematic reviews translated and in accessible language. 1441 followers were registered, 64% women between 25 and 34 years old. Most popular topics: pregnancy, childbirth and breastfeeding. | Evidence synthesis templates or other documents in accessible language | Population | Language  Content  Format | Textual  (on-line) | Implemented and assessed | Need for funding sources for page promotion. | Ease of handling and access to the page, rapid dissemination of content |
| **Educational podcasts from the IHC initiative on key health evidence concepts [Ringle 2020]^119^** | Educational podcasts from the IHC Initiative on key concepts of health evidence in accessible language have been implemented for parents in the United States (called Parents Making Informed Health Choices Podcast). | Teaching/  learning | Population | Language  Content  Format | Verbal | Implemented and assessed | Strategy not tested in people from low-income and community settings. | NR |
| **Communication/ learning about clinical trials**  **[Robinson 2005]^120^** | Clinical trial communication/learning strategies, including key concepts for understanding randomization and equipoise. | Teaching/  learning | Population | Language  Content  Format | Textual | Implemented and not assessed | NR | NR |
| **Evidence synthesis summary template**  **[Rosenbaum 2010]^121^** | Evidence synthesis summary template for communicating the results of systematic reviews to health policymakers in low-income countries (SUPPORT Project, Supporting Policy-relevant Reviews and Trials). | Evidence synthesis templates or other documents in accessible language | Managers | Language  Content  Format | Textual | Implemented and not assessed | Language barrier. | Interaction between managers and collaborators |
| **Use of Facebook and Twitter to communicate evidence in health (DRIFT initiative) [Ryan 2018]^122^** | Creation of a Facebook group (with more than 1,100 members from 41 countries) aimed at parents of children with attention deficit and hyperactivity disorder for communication and dissemination of results of scientific studies on this topic (DRIFT initiative, Disseminating research information through Facebook and Twitter). | Teaching/  learning | Population | Language  Content  Format | Textual  Visual | Implemented and not assessed | Need for funding sources for page promotion. Restricted to one theme. | Ease of access and rapid dissemination of content |
| **Educational podcasts on key health evidence concepts**  **[Semakula 2017^125^ / Semakula 2020^126^]** | Educational podcasts from the IHC initiative on key concepts of health evidence in accessible language were implemented in a group of parents of elementary school students in Uganda. | Teaching/  learning | Population | Language  Content  Format | Verbal | Implemented and assessed | Restricted to similar scenarios.  Long-term effects are not known. | Implemented in low-income country. |
| **Educational podcasts on key health evidence concepts**  **[Semakula 2019b^127^]** | Educational podcasts from the IHC initiative on key health evidence concepts in accessible language. The study describes the evaluation of users. | Teaching/  learning | População | Language  Content  Format | Verbal | Implemented and assessed | Restricted to similar scenarios.  Long-term effects are not known. | NR |
| **Abstract template in accessible language**  **[Santesso 2006]^123^** | Product model with summaries of the content of Cochrane systematic reviews for the population and implemented by the Cochrane Musculoskeletal Group. Strategies include (i) clinical relevance tables with absolute risks, relative risks, and numbers needed to treat for the benefit and harm of the interventions evaluated; (ii) infographics using facial figures to represent the numbers shown in the tables; 100 faces shaded according to the number of people among 100 who benefited or were harmed by the interventions. (iii) abstracts in accessible language and a friendly numerical presentation format. | Evidence synthesis templates or other documents in accessible language | Population | Language  Content  Format | Textual  Visual | Implemented and not assessed | NR | NR |
| **Abstract template in accessible language**  **[Santesso 2015]^124^** | Summary with evidence and results of Cochrane systematic reviews in accessible format and language for the population. The strategy consists of presenting the content in two parts:  (i) narrative summary of the evidence, including an introduction to the concept of a systematic review, background information about the condition and treatment, and information in accessible language about the magnitude of the effect and the certainty of the evidence for relevant outcomes (e.g., “vitamin C is likely to shorten the duration of a cold by a few hours”).  (ii) table with numerical data of the relevant results (absolute effects and confidence intervals presented in frequencies, and information on the certainty of evidence for each outcome, such as symbols and words). | Evidence synthesis templates or other documents in accessible language | Population | Language  Content  Format | Textual  Visual | Implemented and assessed | Content difficult to understand for low-income and/or educational level population. | Interaction between the population and developers |
| **Communication of risks and benefits in health**  **[Sheridan 2003]^128^** | Health risk communication strategies using NNT, RRR, ARR or a combination of these three forms of presentation. | Risk/benefit communication | Population | Language | Textual | Implemented and assessed | Literacy and age group can influence understanding. | NR |
| **Guide for reporting numerical results [The SHARE Approach 2020]^129^** | Guide for communicating numerical health outcomes including, among others, the following recommendations: (i) numbers (1 in 100) rather than nominal terms (many, few); (ii) frequency (9 out of 100) instead of decimal numbers (0.9) or percentages (9%); (iii) same denominator and same follow-up time when comparing numbers; (iv) absolute risk rather than relative risk; (v) use of positive and negative terms (with this treatment, 2 out of 10 people have diarrhea; and 8 out of 10 do not have diarrhea); (vi) use of the metric system understood by the patient; (vii) pie charts for proportions, bar charts for comparing numbers, and line charts for showing change over time. | Guidelines for the development/ assessment of communication products | Population | Language  Content  Format | Textual  Visual | Proposed | NR | NR |
| **Risk communication and other statistical health information**  **[Trevena 2006]^130^** | Strategies and tools for communicating health evidence (risks and other statistical information) to the population. | Risk/benefit communication | Population | Language  Content  Format | Textual  Visual  Verbal | Implemented and assessed | NR | NR |
| **Guidelines for communicating the results of systematic reviews**  **[Welch 2013]^132^** | Guidelines for communicating the results of systematic reviews focusing on equity in health for the population. | Guidelines for the development/ assessment of communication products | Population | Language  Content  Format | Textual | Implemented and not assessed | NR | NR |
| **Guidelines for the elaboration of evidence syntheses in health policies**  **[West African Health Organization 2021]^131^** | Guidelines for the elaboration of evidence syntheses in health policies for managers: (i) define the problem in question; (i) identify and review similar policies; (iii) contextualize the problem; (iv) establish the priorities to be met by the policy; (v) consider acceptability issues; (vi) access and synthesize available evidence; (vii) commission related research; (viii) assess the perception of decision-makers; (ix) organize a technical support committee; (x) organize policy dialogue; (xi) write a policy document; (xii) internally and externally evaluate the proposed policy; (xiii) obtain government approval for the policy, and (ix) monitor and review the policy in question. | Guidelines for the development/ assessment of communication products | Managers | Language  Content  Format | Textual (on-line) | Implemented and not assessed | NR | NR |
| **Health evidence synthesis templates**  **[Wickremasinghe 2016]^133^** | Evidence synthesis model in accessible language to meet the information needs in specific contexts for different audiences. Essential characteristics of each type of synthesis and the ability to respond to specific scenarios were identified. | Evidence synthesis templates or other documents in accessible language | PopulationManagers | Language  Content  Format | Textual | Implemented and not assessed | Technical language level must be appropriate to the target audience level | NR |
| **Guidelines for communicating evidence in health**  **[Woloshin 2003]^134^** | Health evidence communication guidelines considering trhree aspects: (i) clarity: present the risk in units, as “1/1000 in two years” or “10/100 in two years”; (ii) context: communicate the risk rate linked with the patient’s context, considering his/her reality, life expectancy and comparing with people of same age and comorbidities; (iii) uncertainty: present uncertainties associated to the risk estimates presented, including the quality of evidence source, causality criteria and confounders factors. | Guidelines for the development/ assessment of communication products | Population | Language | Textual  Verbal | Implemented and not assessed | NR | NR |
| **Guidelines for communicating evidence in health [Woloshin 2008]^135^** | Health evidence communication guidelines contain the following: (i) What is my risk? - objective: to discuss how to present and contextualize risks; (ii) How can I reduce my risk? - objective: to assess the effect of interventions and the importance of different outcomes; (iii) Does Reduce Risk Have Adverse Events? - objective: consider the adverse effects of interventions and judge whether the benefits outweigh the risks; (iv) How to develop healthy scepticism? - objective: to develop a critical assessment of the literature, including ways to assess conflicts of interest in health studies. | Guidelines for the development/ assessment of communication products | Population | Language | Textual  Verbal | Implemented and not assessed | NR | NR |
| **Guidelines for communicating evidence in health**  **[Woolf 2015]^136^** | Guidelines for communicating evidence in health: (i) specifying the target population; (ii) structure the communication in the form of summary questions and main messages; (iii) determine support methods and tools to facilitate and disseminate knowledge translation. | Guidelines for the development/ assessment of communication products | Population | Language  Content  Format | Textual | Implemented and not assessed | NR | NR |
| **Communication of health risks and benefits with graphics**  **[Zikmund-Fisher 2012]^137^** | Health risks and benefits communication strategies with different types of animated graphics. | Risk/benefit communication | Population | Language  Content  Format | Visual | Implemented and assessed | NR | NR |
| **Guidelines for nominal communication of health benefits**  **[Zikmund-Fisher 2013]^138^** | Guidelines for nominal forms of communication of risks and benefits in health: (i) possibility (may occur, may not occur); (ii) comparative/relative possibility (most likely, least likely); (iii) categorical possibility (high probability, low probability); (iv) relative probability (50% more likely); (v) absolute probability (12% probability); (vi) comparative probability (12% versus 8% probability); (vii) incremental probability (four times more likely). | Guidelines for the development/ assessment of communication products | Population | Language  Content | Textual | Implemented and not assessed | NR | NR |

*AHRQ: Agency for Healthcare Research and Quality; CAPES: Coordenação de Aperfeiçoamento de Pessoal de Nível Superior; CARL: Canadian Association of Research Libraries; CDC: Centers for Disease Control and Prevention; CIHR: Canadian Institutes of Health Research; DECIDE: Developing and Evaluating Communication Strategies to Support Informed Decisions and Practice Based on Evidence;* *DRIFT: Disseminating research information through Facebook and Twitter; ECRAN: European Communication on Research Awareness Need; ERIC: Education Resources Information Center; EVIPNet: Evidence informed policy network; GET-IT: Glossary of Evaluation Terms for Informed Treatment choices; GRADE: IHC: Informed Health Choices; MEDLINE: Medical Literature Analysis and Retrievel System Online; NA: não se aplica (estudo de sínteses incluindo diversas estratégias); NNT: número necessário para tratar; NR: não relatado; REACH-PI: Regional East African community health policy initiative; RRA: redução do risco absoluto; RRR: redução do risco relativo; SORT: Strength of Recommendation Taxonomy; WHO: World Health Organization.*
